# Small Molecule Drugs Targeting Viral Polymerases

**DOI:** 10.3390/ph17050661

**Published:** 2024-05-20

**Authors:** Deborah Palazzotti, Martina Sguilla, Giuseppe Manfroni, Violetta Cecchetti, Andrea Astolfi, Maria Letizia Barreca

**Affiliations:** Department of Pharmaceutical Sciences, University of Perugia, Via del Liceo 1, 06123 Perugia, Italy; deborah.palazzotti@dottorandi.unipg.it (D.P.); martina.sguilla@studenti.unipg.it (M.S.); giuseppe.manfroni@unipg.it (G.M.); violetta.cecchetti@unipg.it (V.C.); andrea.astolfi@unipg.it (A.A.)

**Keywords:** viral polymerase, small molecules, inhibitors, drugs, FDA, viruses

## Abstract

Small molecules that specifically target viral polymerases—crucial enzymes governing viral genome transcription and replication—play a pivotal role in combating viral infections. Presently, approved polymerase inhibitors cover nine human viruses, spanning both DNA and RNA viruses. This review provides a comprehensive analysis of these licensed drugs, encompassing nucleoside/nucleotide inhibitors (NIs), non-nucleoside inhibitors (NNIs), and mutagenic agents. For each compound, we describe the specific targeted virus and related polymerase enzyme, the mechanism of action, and the relevant bioactivity data. This wealth of information serves as a valuable resource for researchers actively engaged in antiviral drug discovery efforts, offering a complete overview of established strategies as well as insights for shaping the development of next-generation antiviral therapeutics.

## 1. Introduction

Over the last four years, the unexpected outbreak of SARS-CoV-2 has pushed researchers throughout the world to work tirelessly to combat the unpredictable COVID-19 pandemic, re-emphasized the critical need to develop novel and effective antiviral drugs to battle new and re-emerging viral threats [1].

In pursuit of this objective, the discovery and development of small molecules specifically designed to target viral polymerases has long been a hot topic. Indeed, these virus-encoded enzymes play a crucial role in viral genome transcription and replication, making them excellent therapeutic targets [2,3,4].

Currently, approved polymerase inhibitors are available for nine human viruses, encompassing herpes simplex virus (HSV), varicella-zoster virus (VZV), human cytomegalovirus (HCMV), hepatitis C virus (HCV), influenza virus (Flu), respiratory syncytial virus (RSV), hepatitis B virus (HBV), human immunodeficiency virus (HIV), and severe acute respiratory syndrome coronavirus 2 (SARS-CoV-2) (Table 1). These viruses can be classified based on their genetic material in DNA viruses (HCMV, HSV, VZV, and HBV) and RNA viruses (HCV, RSV, Flu, Ebolavirus, HIV, and SARS-CoV-2). Among them, three viruses encode for a DNA-dependent DNA polymerase (DdDp)—HCMV, HSV, and VZV—while five utilize an RNA-dependent RNA polymerase (RdRp)—HCV, RSV, influenza virus, Ebolavirus, and SARS-CoV-2. Conversely, HIV and HBV employ an RNA-dependent DNA polymerase (RdDp) for genome replication. While DdDp and RdRp copy the genetic material by using DNA and RNA as template, respectively, RdDp initiates DNA synthesis from RNA, utilizing its retroviral enzymatic activity.

Against this backdrop, we provide here a comprehensive systematic overview of approved small molecules (i.e., organic compounds with a relatively low molecular weight–typically below 1000 Daltons) employed in the treatment of the mentioned viruses through the modulation of viral polymerases. Indeed, while several review articles on this topic are already available in the literature [4,5,6,7,8,9,10,11], to our knowledge, no recent reports have been published that cover all small molecule drugs targeting the viral polymerases of both RNA and DNA viruses with the wealth of information we herein provide.

## 2. Viral Polymerases: An Overview

Virus-encoded polymerases are critical components of viral genome synthesis, which is an intricate process involving numerous host and viral factors. Specifically, this enzyme synthesizes DNA or RNA in the 5′ → 3′ direction by using a parental nucleic acid strand as template to generate a complementary daughter strand. All polymerases share a similar catalytic mechanism, which requires two divalent metal ions (generally Mg^2+^ or Mn^2+^, depending on the specific viral polymerase) coordinated by two/three conserved aspartic acid (Asp) residues [2].

**Figure 1:**
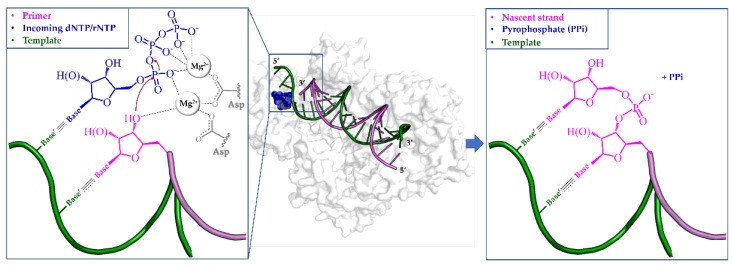
Two-metal ion catalytic mechanism employed by both DNA and RNA polymerases. The primer-dependent mechanism is depicted as an illustrative example (PDB ID 5TXN).

Polymerase reaction involves three consecutive steps: initiation, elongation, and termination. The former stage can be carried out by the viral enzyme using two distinct mechanisms. All DNA polymerases and certain RNA polymerases utilize a primer-dependent mechanism (Figure 1), where the protein adds a deoxyribonucleotide or ribonucleotide triphosphate (dNTP or rNTP, also known simply as NTP) to the free 3′-hydroxyl group (3′-OH) of an appropriate primer, which is a fragment of nucleic acid complementary to the template used as the starting point for replication. Conversely, de novo (primer-independent) initiation is primarily employed by RNA viruses (e.g., HCV polymerase) and does not require a primer, but rather two NTPs. In this scenario, the first NTP (termed mononucleotide primer) furnishes the initial 3′-OH to which a second incoming nucleotide can be linked.

Regardless of the specific initiation mechanism, during the subsequent elongation step, natural nucleotides complementary to the template strand are incorporated into the growing strand through the formation of a new phosphodiester bond. More precisely, the α-phosphate of the incoming NTP is subjected to nucleophilic attack by the 3′-OH of the primer terminus (i.e., the last riboside moiety of the nascent nucleic acid), resulting in the release of inorganic pyrophosphate (PPi) as a byproduct. This continuous process proceeds until either the entire template strand is copied or a termination signal is received.

Notably, the nucleotidyl–transfer reaction is aided by the two catalytic metal ions that stabilize the charge and geometry of the transition state (Figure 1) [12].

Structurally, all viral polymerases share as “cupped” right-hand-like structure composed of three subdomains—known as palm, fingers, and thumb—collaborating to form the binding site for both the DNA or RNA template and the corresponding incoming NTP (i.e., adenosine triphosphate—ATP; guanosine triphosphate—GTP; thymidine or uridine triphosphate—TTP/UTP; cytidine triphosphate—CTP). Notably, the palm domain stands out as the most conserved domain across viral polymerases, housing the catalytic site featuring the three key Asp residues and the two metal ions required for the polymerase enzymatic reaction [13]. The fingers and thumb subdomains, on the other hand, host the template binding region and the NTP binding site, respectively. Additionally, certain viral polymerases may possess supplementary domains to carry out their functions. As an example, HBV and HIV polymerases feature an extra ribonuclease H (RNase H) domain responsible for degrading the RNA strand during the genome replication (Section 4.1.2 and Section 4.2.1). Within this context, it is important to mention that the viral genome replication relies on intricate intermolecular interactions that involve the three palm, fingers, and thumb subdomains, occasionally extending to the other associated domains. [2]

## 3. Targeting Viral Polymerases with Small Molecules

Small molecules targeting viral polymerases constitute a pivotal class of therapeutics designed to combat virus-related infections by precisely disrupting fundamental processes essential to viral replication. Based on their mechanism of action, these compounds are categorized as nucleoside/nucleotide inhibitors (NIs), non-nucleoside inhibitors (NNIs), and mutagenic agents. The forthcoming paragraphs offer a general overview of these two main groups and related subcategories, along with examples of drugs falling into each category.

### 3.1. Nucleoside and Nucleotide Inhibitors (NIs)

This class of small molecule inhibitors competes with the natural NTP for binding to the polymerase’s catalytic site due to their nucleoside- or nucleotide-based chemical structure. To exert the inhibitory effect, NIs must go through a bioactivation process inside the cells. Indeed, these compounds initially enter the cell in a non-phosphorylated (nucleoside) or mono-phosphorylated (nucleotide) form, which then undergoes three or two phosphorylation steps, respectively, to attain the active triphosphate state. Once incorporated into the nascent nucleic acid chain, the NI acts either as an obligate chain terminator or non-obligate chain terminator, thereby impeding the proper synthesis of DNA or RNA. It is worth highlighting that to address issues mainly related to pharmacokinetics (such as poor bioavailability), some NIs have been purposefully designed and developed as prodrugs [14] (Figures 3, 6, 8, 10, 14 and 16).

#### 3.1.1. Obligate Chain Terminators

**Figure 2:**
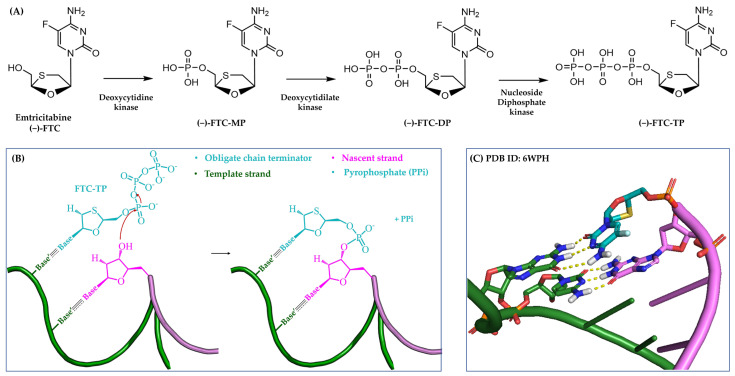
Mechanism of action of obligate chain terminators. (**A**) Cellular bioactivation of the nucleoside-based **(−)-FTC** from the non-phosphorylated to the active form. MP, DP, and TP stand for mono-phosphorylated, di-phosphorylated, and tri-phosphorylated, respectively. (**B**) Simplified 2D representation of the integration of **(−)-FTC-TP** into the nascent strand. (**C**) Three-dimensional (3D) representation of **(−)-FTC** (in cyan) incorporated into the nascent strand (in purple). This image was created using the crystal structure of HIV type 1 (HIV-1) RT in complex with both dsDNA and **(−)-FTC** (PDB ID: 6WPH). Template strand is in green, whereas yellow dotted lines indicate hydrogen-bond interactions.

After the bioactivation procedure, an obligate chain terminator is recognized and incorporated into the nascent nucleic acid as an unnatural substitute for an NTP. This substitution prevents further nucleotides from being added because the inhibitor’s sugar moiety lacks the necessary 3′-OH group crucial for forming the typical 3′,5′-phosphodiester bond [15]. Consequently, the elongation of the growing strand halts, thereby interrupting the synthesis of the viral genome. In Figure 2, the anti-HIV drug **emtricitabine (−)-FTC** (Section 4.2.1) is utilized as a representative example to illustrate the inhibitory mechanism of action employed by obligate chain terminators.

#### 3.1.2. Non-Obligate Chain Terminators

Non-obligate chain terminators closely mimic the chemical structure of natural NTPs, allowing them to be recognized and incorporated by the viral polymerase into the nascent nucleic acid strand. This resemblance also facilitates the subsequent addition of a few more natural nucleotides. Nevertheless, these inhibitors carry modifications or substituents on their nucleobases or sugar moieties which can interfere with post-incorporation events by inducing distortions in the nascent chain, ultimately impairing the elongation efficiency [15,16].

**Figure 3:**
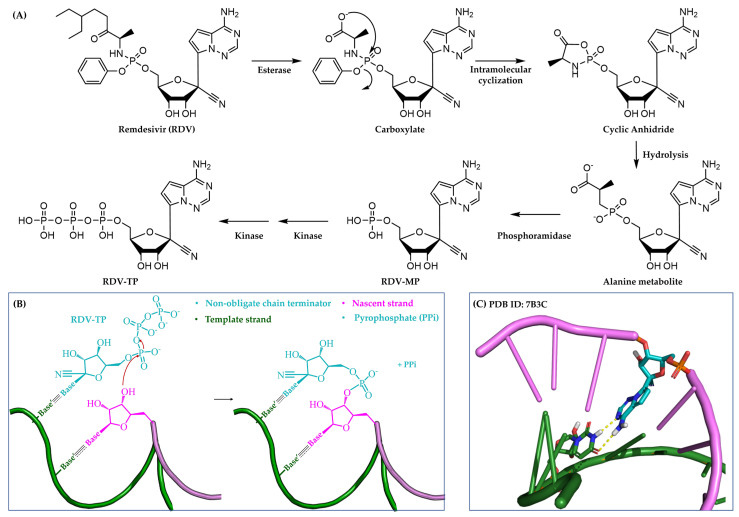
Mechanism of action of non-obligate chain terminators. (**A**) Cellular bioactivation of the prodrug **RDV** to the TP active form. (**B**) Simplified 2D representation of the integration of **RDV-MP** into the nascent strand. (**C**) A 3D representation of **RDV-MP** (in cyan) incorporated into the growing strand (in purple). This image was created using the cryogenic electron microscopy (cryo-EM) structure of SARS-CoV-2 RdRp-RNA-inhibitor complex (PDB ID: 7B3C). Template strand is in green, whereas yellow dotted lines indicate intermolecular hydrogen-bond interactions.

The anti-COVID-19 prodrug **remdesivir** (**RDV**) is the latest FDA-approved non-obligate chain terminator (Section 4.2.5, Figure 3). Recent insights into the molecular mechanism behind **RDV**-induced polymerase stalling revealed the incorporation of other three nucleotides following the inhibitor’s integration, whereas the addition of the fourth nucleotide into the RNA product was hampered by a translocation barrier [17].

### 3.2. Non-Nucleoside Inhibitors (NNIs)

Non-nucleoside inhibitors (NNIs) represent another class of approved drugs that differ from nucleoside and nucleotide analogues in their ability to inhibit viral polymerase without necessitating bioactivation by viral or cellular enzymes. Based on their mechanism of action, NNIs can act as allosteric inhibitors or PPi mimics.

#### 3.2.1. Allosteric Inhibitors

Allosteric inhibitors are a type of drug or molecule that binds to a site on a protein other than the active site, causing a conformational change that reduces the protein’s function. In the specific context of viral polymerase inhibitors, unlike NIs, which exert their action at the catalytic site of viral polymerases, these NNIs target allosteric pockets, inducing conformational changes in the protein that affect polymerase enzymatic function non-competitively.

The most extensively studied antiviral drugs acting with this mechanism of action are those targeting the allosteric site of HIV-1 RT (Section 4.2.1).

#### 3.2.2. PPi Analogues

These NNIs exhibit structural similarity to the inorganic pyrophosphate (PPi) leaving group released during the nucleotidyl transfer reaction (Figure 1). Consequently, their mechanism of action involves the ability to bind the catalytic metal ions at the polymerase’s active site, mimicking the binding pattern of PPi. Currently, the only approved polymerase inhibitor operating via this specific mode of action is the anti-herpetic drug **foscarnet** (**PFA**) (Section 4.1.1), which binds to viral DNA polymerases.

### 3.3. Mutagenic Agents

The mutagenic agents exploit a fundamental weakness in RNA viruses—their error-prone replication process. Unlike DNA viruses, which possess proofreading mechanisms to ensure fidelity during copying, RNA viruses lack this crucial quality control step, and even a slight rise in mutation rates can be enough to push them beyond their extinction threshold. Mutagenic agents capitalize on this vulnerability by mimicking natural nucleotide building blocks used in viral RNA synthesis. Specifically, after biotransformation into their active TP forms, mutagenic agents are mistakenly recognized and integrated by the viral polymerase into the nascent RNA strand. However, due to their structural dissimilarities from natural substrates, these misincorporations create mismatched base pairs, thereby introducing errors into the virus’ genetic code. With each replication cycle, these errors accumulate, creating a cascade of mutations within the viral genome. As the mutation rate surpasses a critical threshold, the virus’s ability to function becomes compromised, leading to the production of non-functional proteins or disrupting critical processes needed for viral replication. In essence, by exploiting the inherent sloppiness of RNA replication, mutagenic agents can exploit an antiviral approach, known as lethal mutagenesis, which aims to push the virus down a mutational path towards extinction [18,19].

Presently, clinically approved drugs reported to act as mutagenic agents include **ribavirin** (**RBV**) (Section 4.2.2 and Section 4.2.3), **favipiravir (FVP)** (Section 4.2.4), and **molnupiravir (NHC)** (Section 4.2.5) [20].

## 4. Small Molecule Drugs Approved to Target Viral Polymerases

In the upcoming paragraphs, we report a comprehensive overview of the 37 small molecules targeting DNA or RNA viral polymerases that have obtained approval as of January 2024 (Table 2). For each licensed drug, details encompassing the targeted virus, the initial approval date, the mechanism of action, and, when available, the specific activity data (such as IC_50_, K_i_ or EC_50_) are provided. Most of the presented biological activities were retrieved from the “NCATS Inxight Drugs” database [21], a comprehensive repository of drug development information available at the following link https://drugs.ncats.io/ (accessed on 30 March 2024). In instances where biological data were unavailable in the specified database, the missing data were retrieved from the relevant literature with associated references provided.

Additionally, to offer appropriate context for each compound, we have included a concise introduction detailing the specific targeted viral polymerase, starting from DNA viruses and then proceeding to RNA viruses.

### 4.1. Small Molecules Targeting DNA Viruses

#### 4.1.1. Herpes Simplex Virus (HSV), Human Cytomegalovirus (HCMV), and Varicella-Zoster Virus (VZV)

Herpes simplex virus (HSV), human cytomegalovirus (HCMV), and varicella-zoster virus (VZV) are members of the herpesvirus family, known for their ability to establish lifelong infections in humans (Table 1). HSV exists in two main types, HSV-1 and HSV-2, which are primarily distinguished by their transmission route and preferred sites of infection. Specifically, HSV-1 is typically transmitted through oral-to-oral contact, causing infections in or around the mouth (e.g., cold sores), whereas HSV-2 is predominantly spread through sexual contact, leading to genital herpes.

HCMV is widespread and can cause severe complications, particularly in immunocompromised individuals and newborns. On the other hand, VZV is responsible for varicella during primary infection and later reactivates to cause herpes zoster.

The DdDp of these viruses share a comparable sequence length of approximately 1200 residues with a primary sequence identity ranging from 30% to 55% [41]. In detail, the protein consists of a catalytic subunit—namely UL30 in HSVs, ORF28 in VZV, and UL54 in HCMV—and a processivity factor, encoded by UL42 (HSVs), ORF16 (VZV), or UL44 (HCMV) genes. Typically, this DNA polymerase performs three different activities: (i) 5′-3′ polymerase activity, crucial for extending the DNA primer; (ii) 3′–5′ exonuclease activity, essential for the proofreading and allowing the excision of mismatched nucleotides from the nascent strand; and (iii) RNAse H activity [42,43].

**Figure 4:**
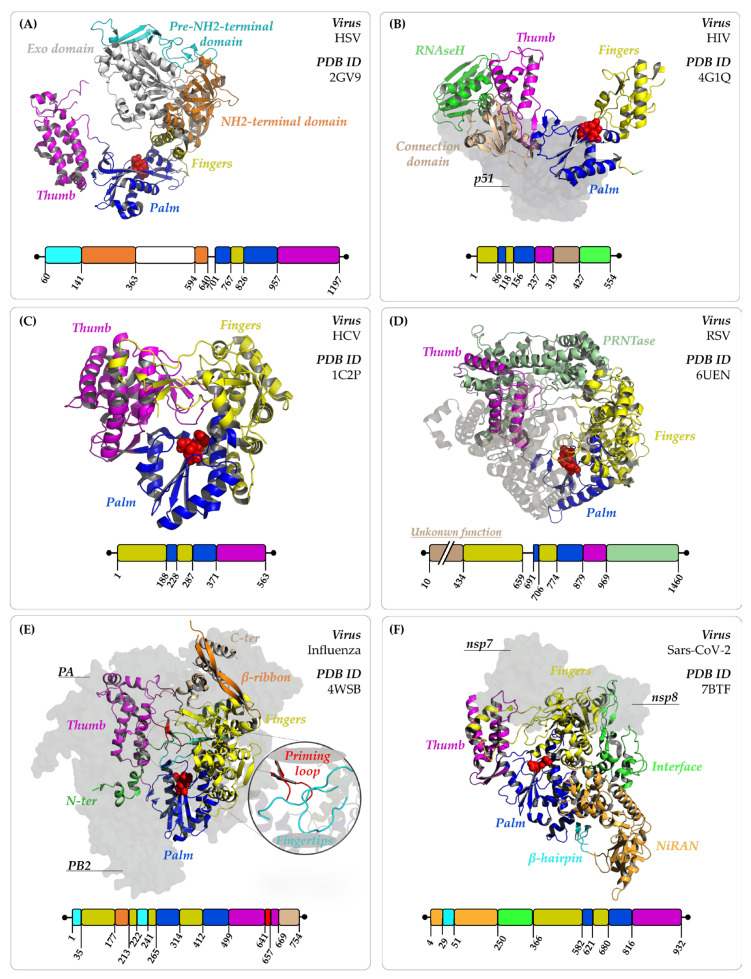
Architectural organization of viral polymerases for which experimental structural information is available. Each panel figure contains the respective PDB ID for reference, along with the 3D structure and corresponding PDB sequence of the polymerase domain. Palm, thumb, and fingers subdomains are illustrated in cartoon and colored as blue, magenta, and yellow, respectively. Catalytic Asp residues are represented as red spheres. (**A**) HSV DdDp. The additional domains Pre-NH2, Exo domain, and NH2-ter domain are showed in white, light blue, and orange, respectively. (**B**) HCV RdRp. (**C**) Influenza polymerase (PB1). The PA (green) and PB2 (grey) subunits are represented as surface. (**D**) RSV RdRp. (**E**) HIV RT. The p51 and p66 subunits are represented as cartoon and surface, respectively. The RNAse H domain is depicted in green, while the Connection Loop is highlighted in wheat. (**F**) SARS-CoV-2 RdRp.

To date, limited (i.e., only a few resolved residues) or no structural information is available for HCMV (UL44-UL54 complex, PDB ID:1YYP) [44] and VZV polymerases, respectively. In contrast, the crystal structure of the catalytic subunit of HSV-1 polymerase (UL30; PDB ID: 2GV9) [45] is publicly accessible on the RCSB Protein Data Bank (RCSB PDB) [46], making it a representative model of this virus family. Specifically, the solved 1197 amino acids (aa) protein is characterized by the pre-NH_2_-terminal, the NH_2_-terminal, and the 3′-to-5′ exonuclease (Exo) domains, along with the palm (residues 701–766 and 826–956), fingers (residues 767–825), and thumb (residues 957–1197) subdomains (Figure 4) [45].

Key structural elements in HSV-1 polymerase compromise the catalytic Asp717, Asp888 and Phe718 residues that coordinate the two catalytic Mg^2+^ ions. Additionally, residues Leu712 and Tyr722 contribute to stabilizing the substrate’s ribose moiety, whereas Arg785, Arg789, and Lys811 regulate the incoming NTP by interacting with the phosphate group, crucially determining the orientation of the phosphate moiety at the 3′-OH of the primer [41].

**Figure 5:**
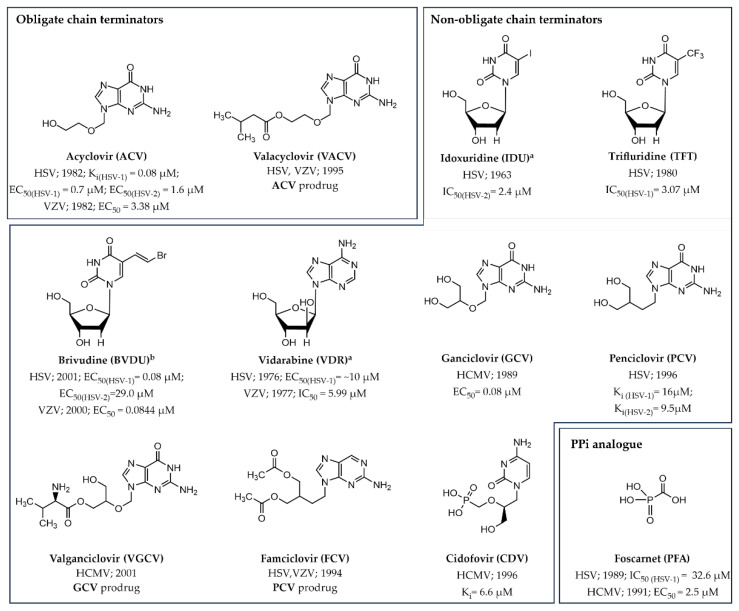
The 2D chemical structures of approved small molecule drugs targeting the herpesvirus polymerase activity. ^a^ Withdrawn from the market and/or no longer recommended for use in the U.S. (https://www.accessdata.fda.gov/scripts/cder/daf/, accessed on 30 March 2024). ^b^ Drugs approved outside U.S.

Moving on to the small molecule drugs, twelve compounds have gained approval for treating the three mentioned herpesviruses (Figure 5) [47,48].

**Figure 6:**
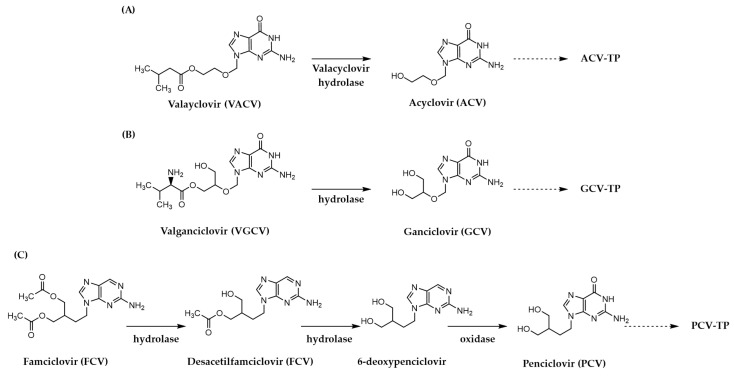
Bioactivation of prodrugs designed to target herpesvirus polymerases for **VACV** (**A**), **VGCV** (**B**) and **FCV** (**C**).

Regarding obligate chain terminator inhibitors, the acyclic guanosine analogue **acyclovir** (**ACV**) is an antiviral drug approved for use against both HSV (HSV-1 and HSV-2) and VZV [22,23,49,50]. In the chemical structure of this inhibitor, the five-membered sugar moiety is replaced by an ether chain lacking the crucial 3′-OH group needed for the phosphotransferase reaction and chain elongation. The oral bioavailability of this NI was then increased via a prodrug strategy, leading to the development of **valacyclovir** (**VACV**), that isthe L-valyl ester derivative of **ACV**, which undergoes in vivo conversion to the parent drug (Figure 6) [51,52].

It is noteworthy that the selectivity of this small molecule drug’s antiviral action and subsequent reduced cellular toxicity stem from the initial phosphorylation process necessary for the inhibitor bioactivation, occurring selectively in virus-infected cells. Indeed, **ACV** is first phosphorylated by virally encoded thymidine kinases and then by cellular enzymes, yielding **ACV-TP** [41].

Along with the two obligate chain terminators described above, nine non-obligate inhibitors targeting the herpesvirus DNA polymerase have been licensed over the years.

The drugs **idoxuridine (IDU)**, **trifluridine (TFT**), and **brivudine (BVDU)** are all 5-halogenated thymidine analogues [24,25,53,54]. **IDU** was the first clinically relevant anti-HSV drug used for the topical treatment of herpetic eye infections [5], although it was later withdrawn from the U.S. around the mid-1990s due to its limited effectiveness and safety concerns, particularly related to eye toxicity and adverse reactions [55].

From a chemical perspective, the substitution of the methyl group at position 5 in the base by an iodine atom alters the steric and electronic characteristics of the compound, enabling it to act as an inhibitor of viral replication [5,54,56]. Differently, the anti-HSV small molecule **TFT** features a trifluoromethyl group at the same position, resulting in a nucleobase of comparable size to that in the natural nucleotide. However, this modification led to considerable decrease in the pKa of the N3 hydrogen, reported as 9.8 and 7.3 for deoxythymidine (dT) and **TFT**, respectively. Consequently, under physiological conditions, only 50% of this NI exists in the non-ionized form, with obvious base-pairing implications [56]. **IDU** and **TFT** are both activated by viral and cellular thymidine kinases, allowing them to be incorporated into the host’s DNA and thereby impacting uninfected cells. As a result, their use is associated with cellular toxicity, limiting their applicability for systemic treatment. Conversely, the 5-(2-bromovinyl) nucleoside analogue **BVDU** displays selective activation solely within cells infected by VZV and HSV-1, possessing a stronger affinity for the viral polymerases [57,58,59]. This heightened selectivity and activity profile stem from specific phosphorylation by the virus-encoded thymidine kinase of the E isomer of **BVDU**, [56,60], which is approximately one hundred-fold more active than the Z form [61,62]. It is worth noting that this NI was approved for use outside United States (U.S.), specifically in a number of European countries including Austria, Belgium, Germany, Greece, Italy, Portugal, Spain, and Switzerland.

The NI **vidarabine** (**VDR**) is distinguished by a structural change that switches the orientation of the sugar hydroxyl group at the C-2 position from the conventional “down” to the “up” position. This simple yet significant modification provides an adenosine analogue by replacing the ribose group with the arabinose unit, rendering this inhibitor highly effective against HSV and VZV [26,27]. Notably, this was the first FDA-approved nucleoside analogue for systemic use in medical settings for herperviruses [63,64,65]. Nevertheless, the primary drawbacks of the drug in clinical applications stem from its restricted solubility in aqueous medium and metabolic instability. As a result, **VDR** is no longer utilized in healthcare settings [5,66,67]. Additional non-obligate inhibitors include the acyclic deoxyguanosine (dG) analogues **ganciclovir** (**GCV**) and its carboisostere analogue **penciclovir** (**PCV**), which are used for the treatment of HCMV and HSV, respectively [10,68,69]. These small molecule chain terminators, unlike the prior acyclic inhibitors **ACV** and **VACV**, retain the crucial 3′-OH group that allows their incorporation into the nascent chain, ultimately leading to the inhibition of viral DNA replication. However, the limited oral bioavailability of **GCV** and **PCV** led to the development of the corresponding prodrugs **valganciclovir** (**VGCV**, mono-L-valyl ester of **GCV**) and **famciclovir** (**FCV**, diacetyl-6-deoxy analogue of **PCV**) (Figure 6), which undergo rapid and extensive conversion to their active forms following oral administration [66,70].

The last licensed non-obligate chain terminator to treat HCMV infections to be mentioned is **cidofovir** (**CDV**) [28], an acyclic nucleoside phosphate (ANP) analogue [71,72,73,74]. In contrast to the other herpesvirus NIs, this deoxycytidine (dC) derivative requires only two phosphorylation steps by cellular kinases to become active due to its inherent phosphonate-mimicking moiety (Figure 6) [75,76].

Structural data regarding any of the mentioned NIs bound to the respective herpesvirus DNA polymerase is currently unavailable. However, it is interesting to note that RCSB PDB houses the crystallographic complex detailing the interaction between **PCV** and the RdRp of SARS-CoV-2 (PDB IDs 7DOK and 7DOI), despite the compound not being authorized for treating this virus (Table 3).

Turning the attention to the NNIs, the sole approved drug to date is **foscarnet** (**PFA**–phosphonoformic acid). This compound serves as a structural mimic of PPi, effectively impeding its release from the natural dNTPs and thus halting the elongation process [77]. Specifically, it has been proposed that **PFA** may impair the shift of the DNA duplex from UL30 catalytic subunit by interacting with the positively charged side chains of the residues R785, R789, and K811 as well as with the two catalytic metal ions [78,79]. Although **PPF** has received approval for treating HSV and HCMV, there is currently no structural evidence demonstrating its binding to the DNA polymerase of herpesviruses. However, the elucidation of drug’s mechanism comes from its co-crystal structure with HIV-1 RT (Table 3, PDB ID 5HP1), which reveals that **PPF** operates by chelating the divalent metal ions within the enzyme’s active site, effectively mimicking the binding pattern of PPi [80].

#### 4.1.2. Hepatitis B Virus (HBV)

Hepatitis B virus (HBV) is a major human pathogen that primarily targets the liver, causing acute and chronic infections. HBV is classified into distinct genotypes labeled A through J, with further subdivision into subgenotypes. These genotypes exhibit geographic variations, being associated with specific regions worldwide, and may entail differences in clinical outcomes, response to treatment, and transmission patterns.

As already mentioned in the introduction, HBV stands apart from other DNA viruses by incorporating a unique reverse transcription step in its replication process. In simplified terms, the viral DNA of HBV serves as a template for the synthesis of an intermediate-RNA product, which subsequently is reverse transcribed into DNA by an RdDp commonly known as reverse transcriptase (RT) [81].

Structurally, HBV polymerase consists of 832 aa organized in four different domains that contribute to its functions (Figure 4). Specifically, these domains include (i) a unique *Hepadnaviridae* terminal protein (TP) crucial for the initiation step of DNA synthesis (i.e., the RNA binding and protein priming); (ii) a spacer domain bridging the TP and RT domains; (iii) the RT domain (~335 aa), which harbors the polymerase activity with palm (residues 50–89 and 173–267), fingers (residues 1–49 and residues 90–172), and thumb (residues 268–351) subdomains; and (iv) a RNase H domain (~155 aa), which cleaves the RNA template during reverse transcription [81,82].

At present, no experimental structural data are available for this polymerase. However, a combination of in vitro studies, such as mutagenesis experiments, and in silico investigations, including sequence comparisons, homology modeling, and molecular dynamics simulations [83], led to the generation of 3D protein models [84,85,86]. Indeed, as HBV and HIV polymerases have a certain degree of sequence identity (~35%), several research teams have undertaken modeling of the HBV enzyme using the known 3D structure of the HIV protein as a template [83,87,88,89]. The most recent 3D protein model of the HBV RT domain was developed using Alphafold program, providing structural insights into this challenging enzyme [86]. Specifically, the RT domain model distinctly revealed the catalytic Asp residues—Asp83, Asp205, and Asp206—responsible for chelating the two crucial Mg^2+^ ions necessary for DNA synthesis, mirroring their positions in the HIV RT (i.e., Asp110, Asp185, and Asp186) [86].

**Figure 7:**
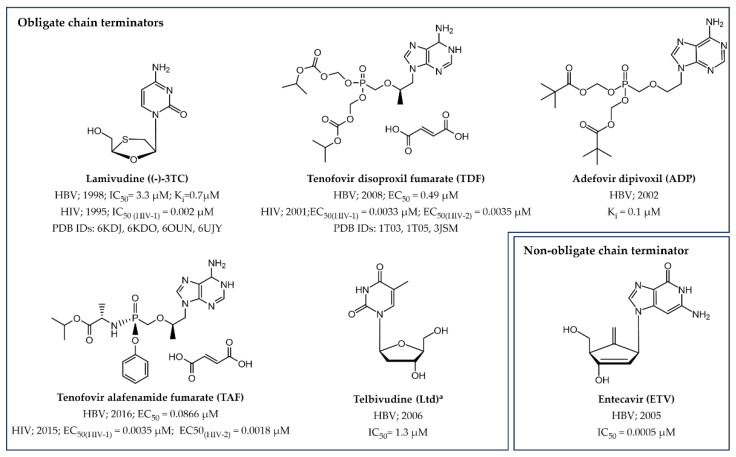
**The** 2D chemical structure of approved NRTIs and NtRTIs inhibiting the HBV polymerase activity. **(−)-3TC** and **TDF** are also licensed as anti-HIV drugs. ^a^ Withdrawn from the market and/or no longer recommended for use in the U.S. (https://www.accessdata.fda.gov/scripts/cder/daf/, accessed on 30 March 2024).

Shifting the focus to the small molecule drugs, presently the FDA has approved three nucleoside and three nucleotide RT inhibitors (NRTIs and NtRTIs, respectively) functioning as DNA chain terminators for the treatment of chronic hepatitis B (CHB) (Figure 7) [90,91].

**Figure 8:**
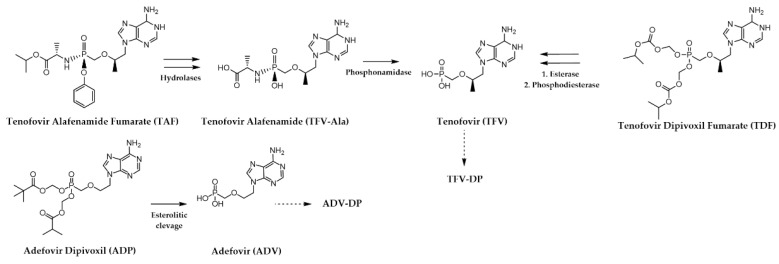
Bioactivation of prodrugs targeting HBV polymerase.

Among the obligate chain terminators, **lamivudine ((−)-3TC)**, a dC analogue characterized by a sulfur atom replacing the 3′ carbon of the ribose became the first licensed oral NRTI for HBV [30]. Thereafter, three ANPs were approved to inhibit HBV replication in infected patients. Specifically, the adenosine derivatives **adefovir dipivoxil (ADP), tenofovir disoproxil fumarate (TDF)**, and **tenofovir alafenamide fumarate (TAF)** were introduced into clinical practice as orally bioavailable prodrugs of the nucleotide analogues **adefovir (ADV)** and **tenofovir (TFV)**, respectively (Figure 8) [31,32,92]. These potent NtRTIs feature a stable phosphonate group, streamlining their activation process to just two phosphorylation steps for conversion into the biologically active diphosphate form. Notably, **(−)-3TC TDF** and **TAF** are successful examples of drug repurposing as they were originally developed for HIV treatment and later repurposed for managing CHB. The last obligate chain terminator NRTI to be named is **Telbivudine** (**Ltd**), the unmodified L-enantiomer of naturally occurring dT [93], which has been discontinued from the US market in 2016 largely for economic reasons and severe side effects [94].

**Entecavir** (**ETV**) stands as the only non-obligate NRTI approved for treating CHB [95,96,97]. This small molecule drug is a dG carbocyclic analogue featuring an exocyclic double bond positioned at the 5′-position. This particular structural element significantly enhances the inhibitor’s affinity and selectivity against the HBV polymerase by effectively filling a hydrophobic binding pocket located at the rear of the RT dNTP binding site [33].

### 4.2. Small Molecules Targeting RNA Viruses

#### 4.2.1. Human Immunodeficiency Virus (HIV)

Human immunodeficiency virus (HIV) is a retrovirus that attacks the immune system, specifically targeting CD4 cells which play a crucial role in the body’s defense against infections. HIV can lead to acquired immunodeficiency syndrome (AIDS), a condition characterized by a compromised immune system, making individuals susceptible to opportunistic infections and certain cancers. The virus has evolved into two major types, HIV-1 and HIV-2, with the former being more prevalent globally. Both are classified into distinct groups and subtypes, reflecting genetic diversity influencing transmission dynamics, regional prevalence, and variations in disease progression and treatment responses.

HIV exhibits a similarity to HBV in the replication mechanism, which is characterized by a reverse transcription process in the viral life cycle [98].

Notably, current understanding of the mechanism of action of retroviral RTs is based on the multiple structural data of HIV-1 RT deposited on RCSB PDB over decades. As of January 2024, an extensive collection of approximately 430 3D structures of this enzyme is accessible, showcasing both wild-type and mutant variants in various catalytic states. In these structures, the HIV RT can be found in its apo form, as well as in association with DNA/RNA and/or inhibitors, providing a comprehensive view of its functional diversity and interactions.

Directing attention to its structural organization, HIV RT is an asymmetric heterodimer composed of two closely related subunits—p66 (560 aa) and p51 (440 aa)—which share a high degree of sequence similarity, highlighted by the identical nature of the first 440 amino acid residues. Nevertheless, these subunits exhibit distinct folding patterns, reflecting their distinct functional roles within the overall RT structure. Specifically, the p66 subunit accommodates the two spatially distinct active sites responsible for the polymerase and RNAse H enzymatic activities within their corresponding domains, while the p51 subunit appears to primarily play a structural role [99].

The polymerase domain is composed of three subdomains: palm (residues 86–117 and 156–236), fingers (residues 1–85 and 118–155), and thumb (237–318) [100,101] (Figure 4). The palm subdomain encompasses three catalytic carboxylates (Asp110, Asp185, and Asp186) that effectively bind two divalent ions (Mg^2+^) crucial for DNA polymerase activity [102]. Other pivotal residues contributing to the polymerase activity include Arg72 and Lys65, both possessing a basic side chain implicated in the binding and stabilization of the β- and γ-phosphates, respectively, in the incoming dNTP [103]. Moreover, Tyr115 participates in the discrimination of incoming nucleotide rNTP and dNTP by functioning as a steric gate in the binding of the deoxyribose ring [104]. The p66 subunit also houses the connection domain (319–426), which acts as a crucial bridge between the polymerase and RNase H domains, facilitating communication and coordination between these two functional regions during the process of reverse transcription.

**Figure 9:**
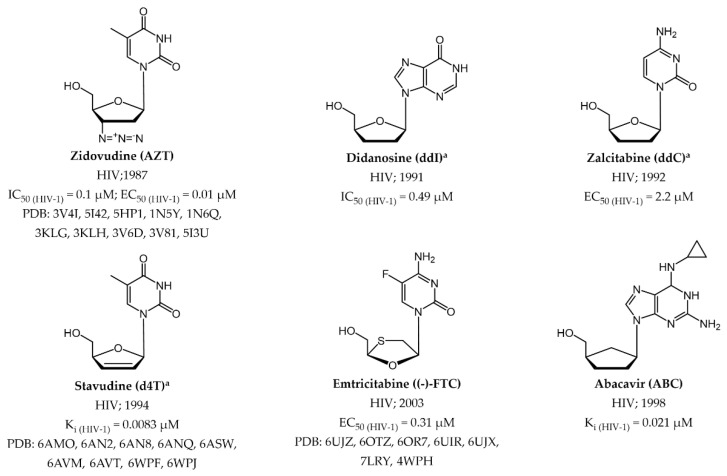
The 2D chemical structure of approved NRTIs and NtRTIs inhibiting the HIV RT polymerase activity as obligate chain terminators. ^a^ Withdrawn from the market and/or no longer recommended for use in the U.S. (https://www.accessdata.fda.gov/scripts/cder/daf/, accessed on 30 March 2024).

Both RT NIs and NNIs have been licenses for treating HIV [105,106]. Regarding NIs, the FDA has authorized seven NRTIs and one NtRTI acting as RNA obligate chain terminators (Figure 7 and Figure 9), which demonstrated a discrete selective activity for the RT enzyme at appropriate concentrations compared to cellular DNA polymerase, owing to their heightened affinity for the viral protein.

**Figure 10:**
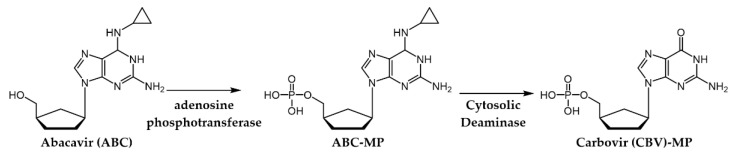
Bioactivation of prodrug Abacavir (ABC) to CBV-MP.

**Zidovudine** (**ZDV**, also recognized as **AZT**) marked the advent of anti-HIV drugs in clinical practice. Structurally, this small molecule is a strict dT analogue where the 3′-OH of the natural substrates was substituted with a bulkier azido group [34]. Subsequently, three more NRTIs were released into the market, wherein the sugar nucleus was replaced by a 2’,3’-unsubstituited 5-member ring. This modification gave rise to the dA analogue **didanosine (ddI)** and dC analogue **zalcitabine (ddC)**, both bearing a tetrahydrofuran moiety, while **stavudine** (**d4T**), functioning as a dT analogue, exhibited a 2’,3’-didehydrofuran ring [35,36]. However, these three compounds were discontinued in the early 2000s mainly due to toxicity and significant side effects (e.g., acute pancreatitis for **ddI** and peripheral neuropathy and lipoatrophy for **ddC** and **d4T**) [107]. Further changes in the sugar nucleus ensued for the dC analogues **lamivudine** (**(-)-3TC)** (Figure 7) and **emtricitabine** (**(-)-FTC)**, which are nucleosides housing a 1,3-oxathiolane ring with an unnatural (−)-stereochemistry [108]. It is worth noting that the only chemical distinction between these two NRTIs lies in the substitution at the 5-position of the of the pyrimidine ring, where, unlike **(−)-3TC**, **(−)-FTC** shows a fluorine atom. Finally, the carbocyclic dG analogue **abacavir** (**ABC**) is the only approved prodrug among NRTIs. Its development successfully addressed the challenges of the parent compound (−)-**carbovir (CBV)** (Figure 10) [109], including low solubility, inadequate oral bioavailability, and cytotoxicity, by introducing a cyclopropyl group at position 6 of the nucleobase [110].

Switching to the NtRTIs, the two ANPs **TDF** and **TAF,** already highlighted among anti-HBV drugs (Figure 7 and Figure 8), are the only nucleotide analogues currently approved for the treatment of HIV infections [111].

**Figure 11:**
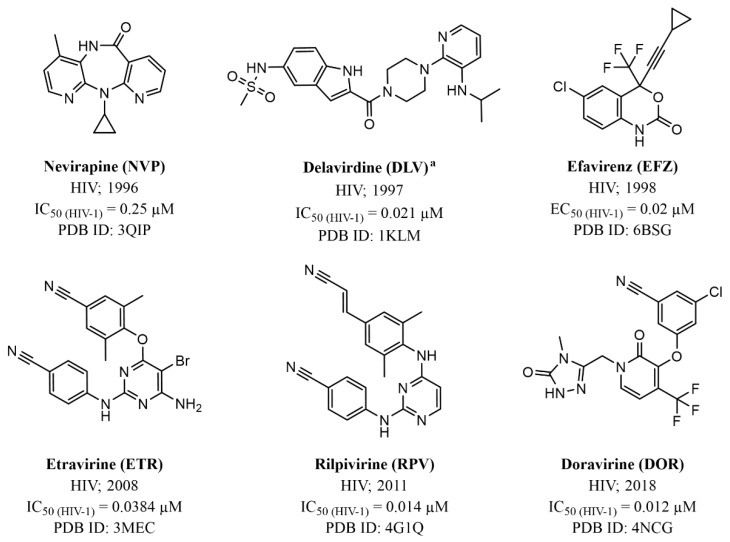
The 2D chemical structure of approved NNRTIs inhibiting the HIV RT polymerase activity. ^a^ Withdrawn from the market and/or no longer recommended for use in the U.S. (https://www.accessdata.fda.gov/scripts/cder/daf/, accessed on 30 March 2024).

In addition to NIs, several allosteric inhibitors, known as non-nucleoside reverse transcriptase inhibitors (NNRTIs), have obtained approval as anti-HIV medications. These include **nevirapine** (**NVP)**, **delavirdine** (**DLV**), **efavirenz (EFZ**), **etravirine** (**ETR**), **rilpivirine** (**RPV**), and **doravirine** (**DOR)** (Figure 11) [105,112].

The introduction of NNRTIs in the 1990s revolutionized HIV treatment. This new class of drugs not only provided new therapeutic options against the virus by working through a distinct mechanism but also boasted a generally better side-effect profile compared to NIs. This translated to improved patient tolerability and adherence, making treatment regimens more manageable.

**Figure 12:**
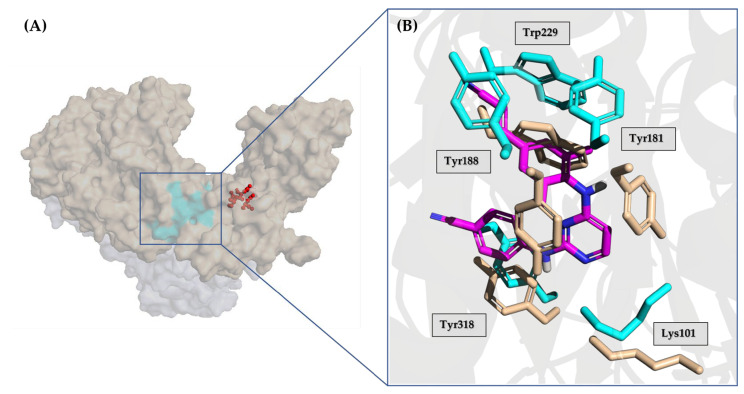
(**A**) Surface representation of the functionally active HIV-1 RT (PDB ID 1DLO [113]). The crucial active site Asp residues are represented by the red ball and stick model, while the surface area representing the allosteric pocket is shaded in cyan. (**B**) Superimposition of two protein states: the functionally active HIV-RT (wheat, PDB ID 1DLO) and the inactive protein conformation (cyan, PDB ID 4G1Q) when bound to the NNRTI **RPV** (purple) [102]. For clarity, only residues situated within 4 Å proximity to **RPV**, along with a RMSD value greater than 2.5 Å between the two protein conformations, are shown.

The NNRTI allosteric binding site resides within the palm domain of the p66 subunit, positioned approximately 10 Å away from the active site of the RT polymerase. This pocket displays hydrophobic characteristics, lined by aromatic (Tyr181, Tyr188, Phe227, Trp229, and Tyr232), hydrophobic (Pro59, Leu100, Val106, Val179, Leu234, and Pro236), and hydrophilic (Lys101, Lys103, Ser105, Asp132, and Glu224) amino acids, alongside two amino acids from the p51 subunit (Ile135 and Glu138). Notably, the allosteric binding site does not naturally exist in the absence of an NNRTI; instead, it is formed due to a drug-induced conformational change in the target, which is particularly noticeable in the side chains of Tyr181, Tyr188, and Trp229 (Figure 12).

As a result of the NNRTI–protein interaction, the catalytic site is blocked in an inactive conformation that is unable to bind the dNTP substrate, hence preventing viral DNA synthesis and virus replication. NNRTIs demonstrate a heightened selectivity for the RT enzyme as they are not substrates for cellular DNA polymerase. This attribute contributes to NNRTIs having a superior therapeutic index and milder adverse effects in comparison to NRTIs and NtRIs.

**Figure 13:**
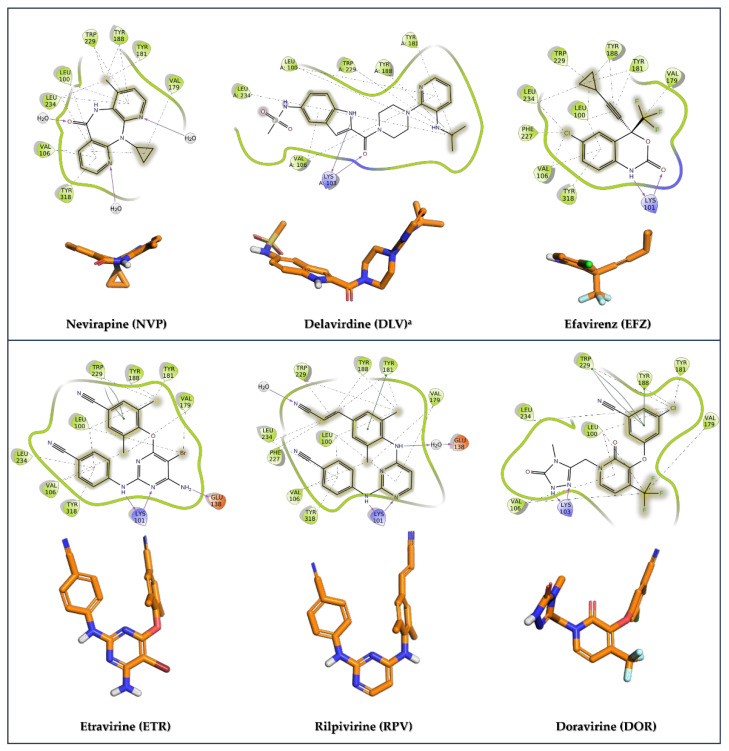
Patterns of 2D ligand–protein interactions and 3D inhibitor conformations extrapolated from crystal complexes of NNRTIs bound to HIV RT polymerase. The intermolecular interactions were derived using software such as the Schrödinger suite 2021-2 (ligand interaction tool) [115] and LigandScout 4.4 [116] and are illustrated as follows: H-bonds (directed or water-mediated) as purple arrows, hydrophobic interactions as dotted lines with the involved ligand portions highlighted in yellow, and π–π interactions as green lines. The 3D ligand-bound conformation for **NVP**, **DLV**, and **EFZ** resemble a butterfly-like structure, while **ETR**, **RPV**, and **DOR** adopt a “horseshoe” or “U” shape. For each drug, the PDB ID with the best resolution has been used as representative. ^a^ Withdrawn from the market and/or no longer recommended for use in the U.S. (https://www.accessdata.fda.gov/scripts/cder/daf/, accessed on 30 March 2024).

**NVP**, **DLV**, and **EFV** belong to the first-generation NNRTIs. They are characterized by a limited conformational flexibility (referred to as a “butterfly-like” structure, Figure 13) that allows the proper filling of the ligand within the HIV-1 RT allosteric pocket. However, this specific conformation also renders these inhibitors highly susceptible to site mutations, which commonly confer cross-resistance among the three NNRTIs [114]. Of note, the manufacturing and distribution of **DLV** was discontinued in the U.S. and Canada mainly for its complex set of interactions with other medications.

The second-generation NNRTIs **ETR**, **RPV**, and **DOR** exhibit a tendency to bind to the viral protein in a “U” shape (also called “horseshoe”, Figure 13) conformation. This characteristic grants them increased structural flexibility, enabling these inhibitors to adapt their binding modes in response to mutations and thus retaining effectiveness against HIV-1 resistance viral strains.

In the context of HIV treatment, it is worth mentioning that N(t)RTIs and NNRTIs are consistently incorporated into antiretroviral therapy (ART) regimens. This comprehensive treatment strategy involves the simultaneous use of multiple antiviral medications that block different stages in the virus’ replication cycle. Indeed, ART typically combines two or more classes of antiretroviral drugs such as RT inhibitors, protease inhibitors, integrase inhibitors, and entry inhibitors. By utilizing a combination of these medications, ART aims to suppress viral replication, minimize the risk of drug resistance, and enhance overall treatment compliance and outcomes for individuals living with HIV [117].

#### 4.2.2. Hepatitis C Virus (HCV)

Hepatitis C virus (HCV) is a blood-borne pathogen responsible for hepatitis C, a liver infection that can lead to severe conditions such as cirrhosis and hepatocellular carcinoma. The virus is classified into different genotypes and subtypes based on genetic variations in its RNA. Currently, there are six major genotypes of HCV, each further divided into multiple subtypes [118].

The HCV RdRp (named NS5B) structure consists of a unique chain (591 residues) that includes the palm (residues 188–227 and 287–370), thumb (residues 371–563), and fingers domains (residues 1–187 and 228–286) (Figure 4). According with the general architecture of the polymerase protein, the active site is placed in the palm subdomain and provides the three Asp residues (Asp318, Asp319, Asp220), coordinating two Mg^2+^ ions during the polymerization reaction [119]. The final 21 amino acids in the HCV NS5B, while not essential for RdRp activity in vitro, play a crucial role as an anchor domain to the membrane, facilitating HCV replication in cell lines [120,121].

In the proximity of the active site, where the palm, thumb, and finger subdomains meet, there exists a hydrophobic region susceptible to interaction with allosteric binding compounds [122]. This area is commonly divided into three palm sites (PS) known as PS-I, PS-II, and PS-III. HCV PS binders could interfere with nucleotide incorporation during the initiation step, consequently impacting RNA synthesis [123]. Furthermore, two additional allosteric sites, named thumb site (TS) I and II, are present within the thumb domain. Inhibitors targeting these specific sites possess the capacity to interfere with the protein conformational changes that occur during the transition from the initiation to the elongation steps in the replication process [124].

**Figure 14:**
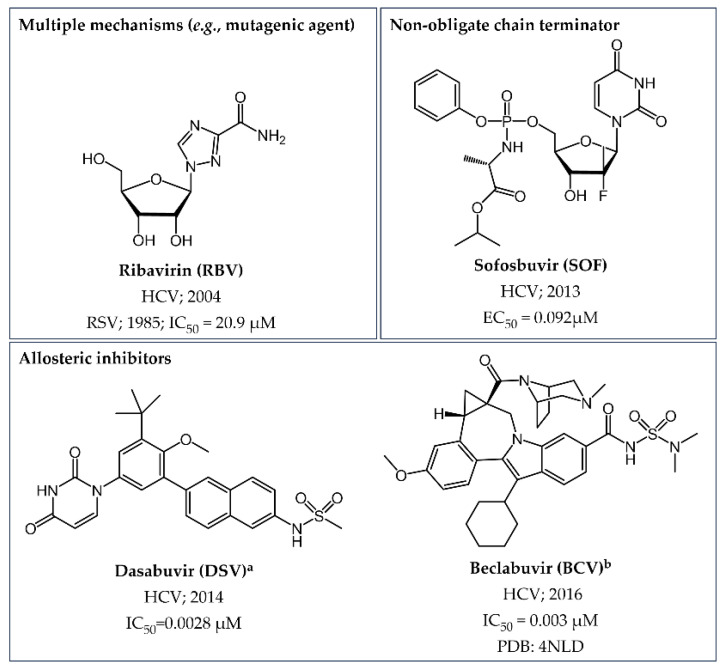
The 2D chemical structures of approved small molecule drugs targeting the HCV polymerase. ^a^ Withdrawn from the market and/or no longer recommended for use in the U.S. (https://www.accessdata.fda.gov/scripts/cder/daf/, accessed on 30 March 2024). ^b^ Drugs approved outside U.S.

The first small molecule approved for the treatment of HCV infections was **Ribavirin** (**RBV**) (Figure 14), which had previously received authorization as antiviral drug for addressing RSV infections (Section 4.2.3). The mechanism of action of this triazole carboxamide derivative is still under debate, with various hypotheses proposed, ranging from indirect (e.g., the inhibition of inosine monophosphate dehydrogenase and immunomodulation) to direct (e.g., the inhibition of RNA capping and RdRp activities) effects on viral survival and replication [125]. In particular, one theory suggests that **RBV** may function as a mutagenic agent targeting viral polymerase. Operating in this manner, **RBV** acts as a nucleoside analogue structurally resembling guanosine. Upon administration, the drug undergoes phosphorylation within the host cells to form **RBV-TP,** which can be incorporated into the growing RNA chain during viral replication. Remarkably, this drug can form base pairs with both cytosine and uracil. The ensuing mismatch incorporation introduces a cascade of mutations into the viral genome, ultimately hindering the virus’ ability to function and replicate effectively. However, it is important to note that the antiviral activity of **RBV** as a standalone therapy is not sufficient for effective HCV treatment. Typically, this drug is combined with other antiviral agents for a more comprehensive therapeutic approach [126].

Indeed, the advent of new medications named direct-acting antivirals (DAAs) has revolutionized the treatment landscape for HCV, providing potent therapeutic solutions for effective cure and long-term complication prevention. Specifically, one NI and two NNIs were introduced into the market as anti-HCV drugs (Figure 14).

**Figure 15:**
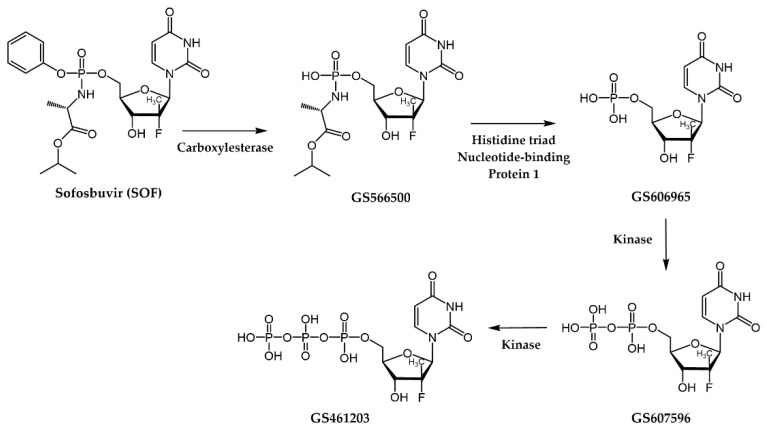
Bioactivation of prodrug **SOF** to the pharmacologically active form **GS461203**.

**Sofosbuvir (SOF)**, a breakthrough in the treatment of HCV infection, is a NI acting as a non-obligate chain terminator [38]. Particularly, **SOF** is a nucleotide prodrug that undergoes intracellular metabolism to form its active triphosphate form (**GS461203**, Figure 15). Structurally, **SOF** mimics deoxyuridine (dU) but distinguishes itself by incorporating a methyl group along with a fluorine atom at the 2′-position of the ribose ring instead of a hydroxyl group. This modification substantially boosts the drug’s stability against enzymatic degradation, allowing for prolonged intracellular half-life and improved efficacy. Often used in combination with other antiviral agents, **SOF** has demonstrated high cure rates across various HCV genotypes and has substantially transformed the HCV therapy field due to its efficacy, tolerability, and relatively low incidence of adverse effects. It is noteworthy, that the PDB ID 4WTG [127] provides valuable structural information about the mechanism of action of **SOF** by revealing the details of the ternary complex involving NS5B, an RNA primer, and its diphosphate metabolite **GS607596** (Figure 15). More precisely, this 3D structure aids in understanding how **SOF’s** modifications at the 2′-position impede the function of the HCV NS5B polymerase by inducing steric clashes, disrupting key hydrogen bonds, and ultimately stalling the polymerase complex, thereby inhibiting viral RNA replication.

**Figure 16:**
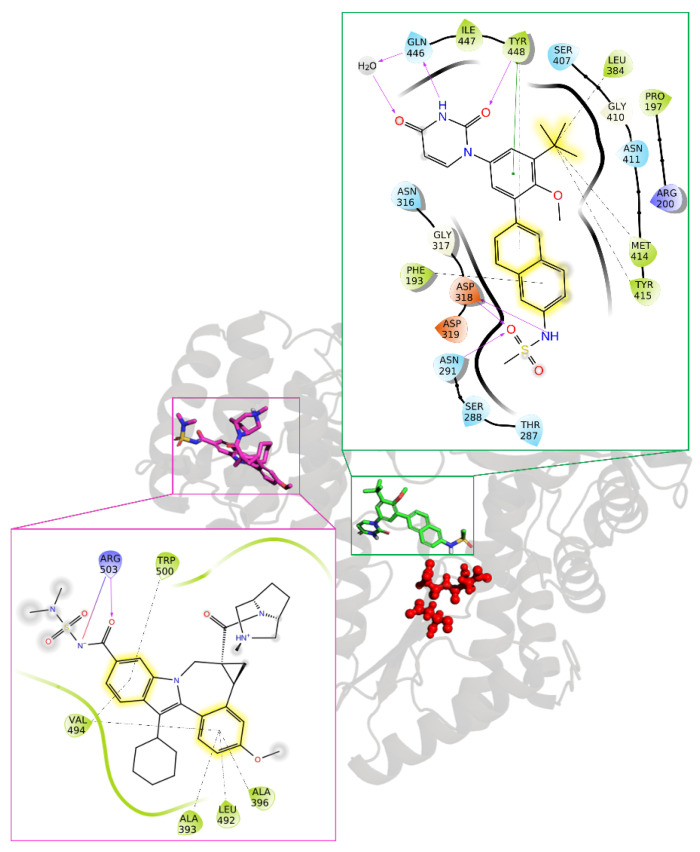
HCV NS5B with approved NNIs binding to distinct allosteric sites. Specifically, **DSV** (green) occupies the PS-I, while **BCV** (magenta) resides within the TS-I. The experimental position of **BCV** is derived from the PDB ID 4NLD [130], while the **DSV** location was generated by SeeSAR 13.0.1 [131] molecular docking using its analogue binder in the PDB ID 4MKB [129] as reference compound. The intermolecular interactions were derived using software such as the Schrödinger suite 2021-2 (ligand interaction tool) [115] and LigandScout 4.4 [116] and are illustrated as follows: H-bonds (directed or water-mediated) as purple arrows, hydrophobic interactions as dotted lines with the involved ligand portions highlighted in yellow, and π–π interactions as green lines Key catalytic residues Asp318, Asp319, and Asp220 are highlighted in red using a ball and stick representation.

Turning our attention to the NNIs, **Dasabuvir** (**DSV**) stands out as the sole FDA-approved allosteric NNI for combating HCV. The approval of **DSV** marked a significant advancement in HCV treatment, especially in combination therapies with other DAAs, contributing to improved cure rates and offering a more effective treatment option for individuals infected with HCV genotype 1 [128]. Indeed, its specificity for inhibiting the HCV genotype 1 NS5B polymerase is impressively high, being at least 7000 times more selective for this target over human/mammalian polymerases. This NNI operates by targeting the allosteric PS-I within the NS5B, strategically located close to the catalytic region. By binding to this specific site, **DSV** induces a significant protein conformational change, disrupting the enzyme’s ability to elongate the viral RNA strand during replication. Structural insights into the mode of action of this drug are unveiled through the crystallography complex of its strict analogue bound to HCV NS5B polymerase (PDB ID 4MKB) (Figure 16) [129]. However, this drug has been voluntarily discontinued by the manufacturing company for strategic business reasons, as it has been replaced by more effective and better tolerated options.

**Beclabuvir** (**BCV**) is the second NNI which has gained approval for use in combination with other DAAs but only outside the U.S., specifically in Japan. Co-crystal data (PDB ID 4NLD) revealed that, in contrast to **DSV**, this allosteric inhibitor binds to the TS-I (Figure 16), a pocket situated approximately 30 Å away from the catalytic NS5B RdRp site. Consequently, **BCV** hinders the formation of a productive RNA–enzyme complex, thereby disrupting the viral RNA replication process [122].

#### 4.2.3. Respiratory Syncytial Virus (RSV)

Respiratory syncytial virus (RSV) is a common respiratory pathogen that primarily affects infants and young children, causing respiratory tract infections such as bronchiolitis and pneumonia.

The RSV RdRp is a complex machinery composed of several essential components that work together to accomplish the replication and transcription of the viral RNA genome. At the epicenter of this intricate system lies the large polymerase (L) protein, an architectural behemoth comprising 2165 aa (~1460 aa in the available solved cryo-EM structures) [132]. This pivotal protein is intricately organized into five distinctive structural and functional domains, each delineated by specific residues that play a crucial role in executing the enzymatic activities. Specifically, the polymerase domain spans residues 1–968 and forms the central catalytic core responsible for RNA replication. It encompasses hallmark subdomains—the palm (residues 692–705 and 774–878), fingers (residues 434–659 and 706–773), and thumb (residues 879–968)—each contributing to catalysis and RNA binding. The catalytic motif, Gly810-Asp811-Asn812q, along with Asp700, coordinates two requisite Mg^2+^ ions required for phosphodiester bond formation [133]. Ranging from residues 969 to 1460, the polyribonucleotidyl transferase (PRNTase or capping) domain governs crucial capping processes by adding a protective chemical group (cap) to the newly synthesized viral RNA transcripts. The third functional domain of the L protein is the unsolved methyltransferase (MTase) domain, which catalyzes the methylation of the capped RNA molecules [134] (Figure 4). Besides these three enzymatic domains, the RSV L also contains two unsolved structural domains, named the connector domain (CD) and the C-terminal domain (CTD), which work in conjunction to support the overall architecture of the polymerase complex. However, the L protein cannot function on its own and requires a helper protein, phosphoprotein (P), to be fully active. Together, the L–P complex is responsible for the crucial steps of viral RNA synthesis and modification, essential for RSV replication.

As of now, the RCSB PDB hosts six detailed cryo-EM structures which offer a detailed visualization of the overall architecture and organization of the RSV polymerase complex (PDB IDs: 6UEN [132]; 6PZK [134]; 8FPI [135]; 8FU3 [136]; 8SNX; 8SNY [137]). Specifically, these structures elucidate how the L and P proteins interact, their relative positioning, and the arrangement of domains within the polymerase assembly.

When delving into antiviral treatments, to date the only small molecule drug authorized by the FDA to treat RSV is the already mentioned anti-HCV agent **RBV** (Section 4.2.2) (Figure 14) [37]. Specifically, **RBV** acts as a mutagenic agent and hampers RSV replication. Notably, the early administration of **RBV** during the onset of infection has demonstrated clinical benefits, particularly in children [138].

#### 4.2.4. Influenza Virus

Influenza, commonly known as the flu, is a highly contagious respiratory infection caused by a dynamic group of viruses categorized into types A, B, C, and, sporadically, D. Among them, influenza A holds prominence due to its ability to mutate and adapt, birthing diverse strains with the potential to provoke widespread outbreaks.

The influenza A RdRp is a heterotrimeric complex of about 2300 amino acids, composed of three subunits: the polymerase basic protein 1 (PB1, 756 aa), the polymerase basic protein 2 (PB2, 760 aa), and the polymerase acidic protein (PA, 713 aa). The tight association between these three essential subunits and their correct assembly allows the viral polymerase to accomplish the precise and controlled replication and transcription of the viral RNA genome [139,140,141].

The high-resolution X-ray (e.g., PDB IDs 4WSB and 5M3H) and cryo-EM (e.g., 6SZU) structures of RdRp from the different influenza virus types provided significant understanding regarding the replication mechanism and the structural architecture of the polymerase subunits.

Taking influenza A as an example, the PB1 is the catalytic core of the RdRp complex, possessing the enzymatic activity for RNA synthesis. Structurally, PB1 accommodates the conserved polymerase palm (residues 265–313 and 412–498)—housing the catalytic Asp residues (Asp305, Asp444 and Asp445) along with two Mg^2+^ ions—as well as the thumb (residues 499–668) and fingers (residues 35–264 and 314–411) subdomains [139]. PB1 interacts with both PB2 and PA subunits, which are involved in cap-snatching and endonuclease activity, respectively. Additionally, this subunit contains supplemental segments (i.e., N- and C-terminal extensions, NLS β-ribbon, fingertips and priming loops; Figure 4), which facilitate PB1’s interaction with the nucleic acid and other components of the replicative complex [139].

Notably, the influenza virus RdRp operates in concert with other viral elements. Specifically, it forms a functional partnership with the viral RNA (vRNA) genome, a process facilitated by the binding of another viral protein known as nucleoprotein (NP). The intricate collaboration of RdRp, vRNA, and NP results in the formation of a cohesive and dynamic unit referred to as the viral ribonucleoprotein (vRNP) complex.

**Figure 17:**
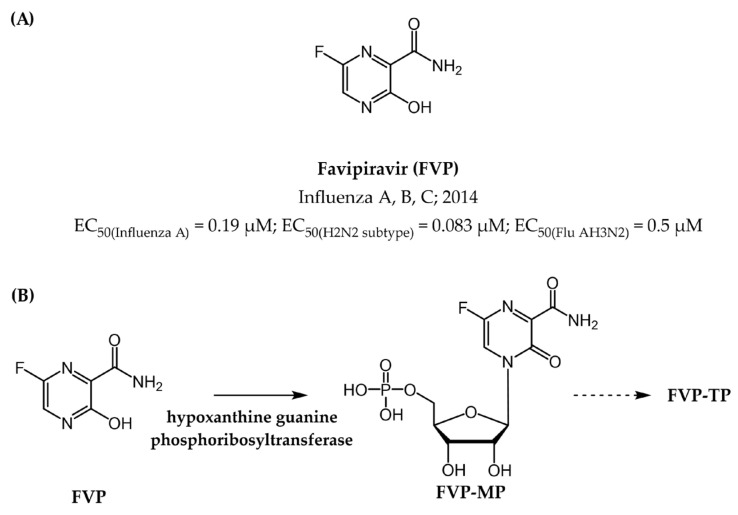
(**A**) The 2D chemical structure of the only approved small molecule targeting the influenza polymerase by multiple mechanisms (e.g., as mutagenic agent). It should be noted that this drug was approved outside the U.S. (**B**) Bioactivation of prodrug **FVP**.

Moving on to small molecule drugs, the only licensed agent which targets the polymerase activity of the RdRp influenza viruses is the prodrug **favipiravir** (**FVP**), a pyrazine carboxamide derivative (Figure 17). Notably, this approval is exclusive to Japan for use against emerging influenza viruses resistant to other antivirals [142,143].

Multiple mechanisms underpinning the action of **FVP** have been proposed, including non-obligate chain termination, slowed RNA synthesis, and the induction of lethal mutagenesis [144,145,146]. In the latter hypothesis, the active form, **FVP-TP**, competes with the purine nucleotides ATP and GTP and is erroneously incorporated into the newly forming viral RNA strand by the RdRp enzyme [147]. Following integration, this drug exhibits promiscuous base pairing with cytidine and uridine, resulting in an elevated mutation rate within the viral genome. This accumulation of mutations ultimately leads to the production of nonviable viral progeny, effectively hindering viral replication and propagation within the host [145,148].

#### 4.2.5. Severe Acute Respiratory Syndrome Coronavirus 2 (SARS-CoV-2)

Severe acute respiratory syndrome coronavirus 2, commonly known as SARS-CoV-2, emerged as the causative agent of the global pandemic known as COVID-19. This novel coronavirus first came to public attention in December 2019 when a cluster of pneumonia-like cases was reported in the city of Wuhan, Hubei province, China. The virus quickly disseminated worldwide, leading to widespread illness, significant disruptions to daily life, and profound impacts on public health systems and economies globally [149,150,151]. One of the defining features of SARS-CoV-2 is its ability to cause a range of respiratory symptoms, from mild cold-like symptoms to severe pneumonia and acute respiratory distress syndrome (ARDS), particularly in older adults and individuals with underlying health conditions. Asymptomatic cases and presymptomatic transmission have further complicated efforts to control the spread of the virus [152,153,154,155].

The rapid spread of SARS-CoV-2 and the resulting pandemic outbreak necessitated an urgent quest for identifying small molecules with the potential to fight this deadly virus [156,157,158]. In response to this challenge, substantial drug discovery efforts have been directed towards the RdRp complex as a therapeutic target. It is noteworthy that since the first cryo-EM structure was released in April 2021 (PDB ID: 6M71) [159], a total of 48 complex structures, depicting different protein functional states along with various cofactors and inhibitors, have been disclosed on RCSB PDB as of January 2024 [160,161,162]. This wealth of structural information substantially contributes to our comprehension of the viral polymerase and the intricate mechanisms governing virus’s life cycle.

SARS-CoV-2 viral replication machinery comprises several non-structural proteins (nsp), among which nsp12 corresponds to the viral RdRp (Figure 4). Generally, nsp12 does not act independently, but it interacts with nsp7 and nsp8, collectively forming the “minimal core component” crucial for RNA synthesis. This intricate arrangement enables the RdRp complex to achieve maximum replication efficiency [163,164]. However, the complete assembly of the replication macro-complex requires additional nsp subunits, including the cofactors nsp9 and nsp10, the helicase nsp13, the proofreading exonuclease and methyltransferase nsp14, and the methyltransferase nsp16 [165].

The overall layout of nsp12 encompasses 932 aa structured into a multi-domain protein. Within this architecture, nsp12 exhibits a conserved organization featuring a C-terminal RdRp domain (residues 366–932), playing the pivotal role in catalyzing the formation of phosphodiester bonds among ribonucleotides in the presence of divalent metal ions. The conserved active site resides within the palm domain (residues 582–620 and 680–815), housing the catalytic Asp residues corresponding to Asp760, Asp761 [159]. Additionally, the fingers subdomain (residues 366–581 and 621–679) assists in establishing the active site geometry by securely holding the RNA template, while the thumb subdomain (residues 816–932) aids in stabilizing the initiating NTP on the template [164].

On the other hand, the N-terminal region contains a nidovirus-specific domain (NiRAN, residues 4–28 and 51–249), which is essential for replication owing to its nucleotidyltransferase activity [159,166]. The polymerase and the NiRAN domains are connected by an interface domain (residues 250–365). An additional N-terminal β hairpin domain fits into the groove clamped by the NiRAN and the polymerase palm domains.

**Figure 18:**
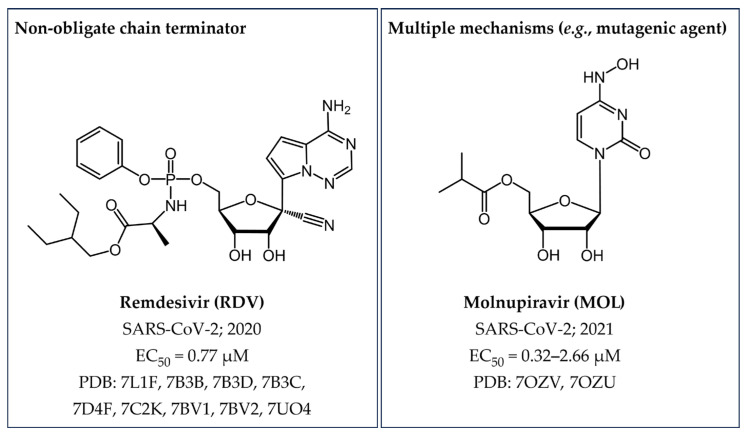
The 2D chemical structure of approved small molecules targeting the SARS-CoV-2 polymerase.

In the realm of approved drugs targeting SARS-CoV-2 RdRp, there are two nucleotide analogues acting with a distinct mechanism of action (Figure 18).

**Remdesivir** (**RDV**) is a broad-spectrum antiviral small molecule demonstrating efficacy in inhibiting the RdRp across various RNA viruses (e.g., HCV, SARS-CoV, and MERS-CoV), including SARS-CoV-2 nsp12 [39,167]. Indeed, on May 2020 this intravenously administered small molecule, originally developed to combat HCV, was the first NI receiving emergency use authorization (EUA) and subsequent approval from regulatory agencies for treating both adult and pediatric COVID-19 patients [168,169,170]. Chemically, **RDV** is the mono-phosphoramidite prodrug of a 1′-cyanoadenosine analogue. As highlighted in Section 3.1.2, **RDV** acts as a non-obligate chain terminator (Figure 3) due to the strategic presence of a CN group at the C1’ position in its chemical structure, which introduces a steric hindrance around the 3′-OH group, thereby disrupting the proper positioning of subsequent nucleotides and effectively halting RNA chain elongation [17,171].

**Figure 19:**
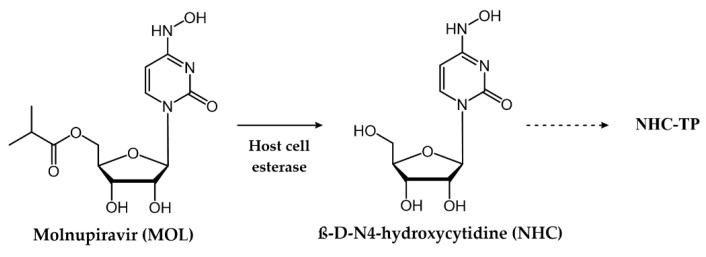
Bioactivation of prodrug **MOL**.

**Figure 20:**
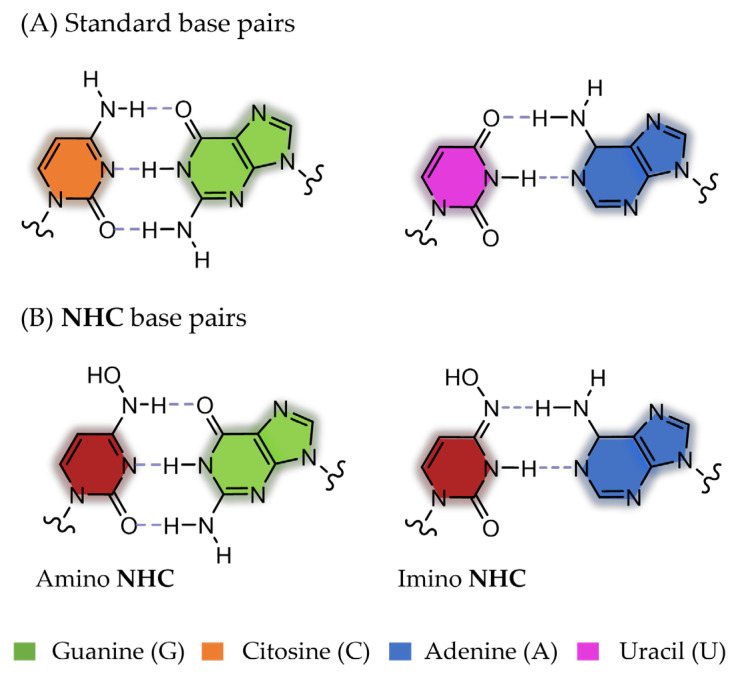
Schematic illustration of the base pairs involving standard nucleobases (**A**) and the two different tautomeric forms of **NHC** (**B**), represented in red, in the RdRp active center.

The mutagenic agent **Molnupiravir** (**MOL**), also known as **EIDD-2801** or **MK-4482**, marked the milestone as the first oral antiviral sanctioned for COVID-19 patients [172] (Figure 18). In December 2021, the FDA issued an EUA for **MOL**, specifically for the treatment of mild-to-moderate COVID-19 in adults who have tested positive for direct SARS-CoV-2 viral infection and are at high risk of progressing to severe illness, such as hospitalization or mortality [40,173,174]. This authorization was applicable when FDA-authorized alternative treatment options for COVID-19 were either inaccessible or clinically unsuitable. Chemically, **MOL** represents the isopropyl ester prodrug of the ribonucleoside analogue **β-D-N4-hydroxycytidine (NHC)**, also named **EIDD-1931** (Figure 19). This small molecule drug exhibits its antiviral activity against SARS-CoV-2, through a mechanism involving the induction of lethal mutagenesis. Upon oral administration, the metabolite **NHC** is converted into the active form **NHC-TP**, which is mistakenly recognized as either a CTP or UTP building block by the RdRp [175]. Indeed, one of the intriguing aspects of this drug is its ability to exist in the two distinct amino or imino tautomeric forms (Figure 20). The former, akin to cytosine, pairs with guanine, while the latter, resembling uracil, pairs with adenine. This flexibility in base pairing introduces ambiguity during the copying process, inducing a high frequency of mutations within the viral genome and, ultimately, causing the production of nonviable viral particles [176].

**Figure 21:**
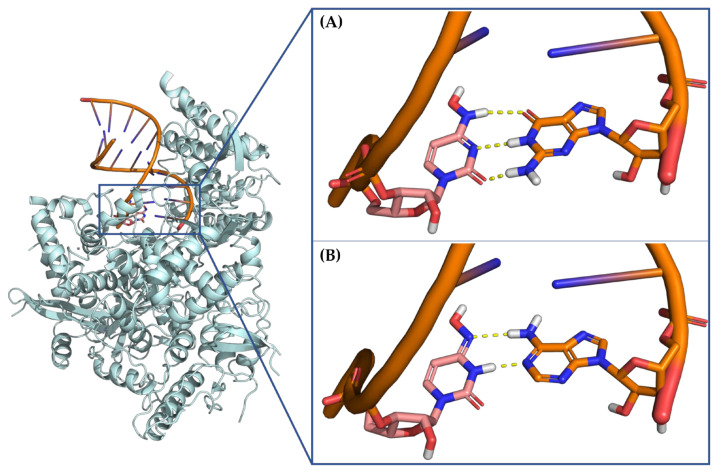
SARS-CoV-2 RdRp complexed with the two tautomeric forms of **NHC**. The amino **NHC** and the imino **NHC** are base-paired with G (PDB ID: 7OZV) (**A**) and A (PDB ID: 7OZU) (**B**), respectively. Hydrogen bonds between **NHC** and G/A are represented as dashed yellow lines.

Notably, the availability of 3D structural models depicting **NHC** integrated into the RNA template strand enhanced our understanding of how this mutagenic agent engages with its target at the atomic level, contributing to a more thorough comprehension of its antiviral action [177]. Indeed, two structures of SARS-CoV-2 RdRp, featuring stable **NHC** g and **NHC**-A base pairs within the enzyme’s active site, have been deposited on RCSB PDB with the IDs 7OZV and 7OZU, respectively (Table 3) (Figure 21) [175].

## 5. Conclusions

The discovery and development of small molecule drugs targeting viral polymerases has yielded remarkable successes in combating viral diseases. One of the most notable successes in this field has been the development of NRTIs and NNRTIs for the treatment of HIV/AIDS (Section 4.2.1). These small molecule drugs have transformed the management of HIV, allowing patients to achieve long-term viral suppression and improved quality of life. Similarly, the development of DAAs targeting HCV polymerase has revolutionized the treatment of chronic hepatitis C (Section 4.2.2), with cure rates exceeding 95% in many cases.

Nevertheless, the fight against these ever-evolving threats persists, presenting several challenges and opportunities that remain to be tackled, the most crucial of which are outlined below.

Viral polymerases can rapidly acquire mutations that confer resistance to existing drugs, rendering them less effective or even ineffective [178]. This challenge is especially pronounced in RNA viruses (Table 1), which exhibit high mutation rates and a remarkable ability to rapidly adapt to selective pressures. Consequently, this presents a significant threat that requires extensive research efforts to investigate diverse strategies aimed at designing and developing next-generation small molecule inhibitors with heightened potency against drug-resistant viral strains. This involves, but is not limited to, the use of high-throughput screening technologies and advanced computational modelling/artificial intelligence methods to identify novel chemical scaffolds and refining existing inhibitors for improved resistance profiles [118,179,180,181,182]. Strategies may include, for example, targeting conserved regions of the enzyme that are less prone to mutation or designing molecules with increased structural flexibility (as demonstrated by second-generation HIV NNRTIs compared to their first-generation counterparts), the latter being able to dynamically adjust their binding modes in response to mutations, thereby preserving their efficacy against viral strains that have acquired resistance.

The current arsenal of small molecule drugs, though effective in providing targeted treatment against specific viruses, offers limited broad-spectrum coverage. This necessitates the development of pan-viral inhibitors capable of effectively combatting a wider range of viral threats [183,184]. Such drugs show several advantages over drugs with narrower specificities. Firstly, broad-spectrum inhibitors have the potential to treat patients with co-infections caused by multiple viruses simultaneously, reducing the risk of drug interactions and simplifying treatment regimens. Secondly, pan-viral drugs can serve as a crucial first line of defense against emerging or re-emerging viruses with epidemic and/or pandemic potential (Table 4), providing proactive therapeutic options before specific treatments are developed. In the context of the pharmacological target herein discussed, as different viral polymerases can share structural and functional similarities, small molecules designed to target one type of viral polymerase can be effective against others. By leveraging this universality among viral polymerases, researchers can advance the development of innovative antiviral therapies that offer broader coverage and greater effectiveness in combatting viral infections, thereby addressing the challenges posed by viral diversity and the continual emergence of new viral strains. Notable examples of broad-spectrum antiviral drugs targeting viral polymerases include: (a) **ribavirin** (Figure 14), which exhibits activity against various RNA viruses such as HCV, RSV, and Lassa fever virus; (b) **remdesivir** (Figure 18), known for its antiviral effects against Ebola virus, coronaviruses (including SARS-CoV-2), HCV, and RSV; and **favipiravir** (Figure 17), effective against influenza, Ebola virus, Lassa virus, and Severe fever with thrombocytopenia syndrome virus (SFTSV) [142,144,185].

Finally, there is a pressing need for the optimization of combination antiviral therapy [180,181,186,187]. This imperative arises from the complexity and adaptability of viruses, which can develop resistance to single-drug treatments over time. By employing combinations of drugs with distinct mechanisms of action, akin to the approach used in ART for HIV (Section 4.2.1), researchers can target multiple stages of the viral life cycle simultaneously, increasing the likelihood of suppressing viral replication and reducing the emergence of drug-resistant strains. Moreover, leveraging synergistic interactions between different antiviral agents has the potential to enhance overall treatment efficacy. However, optimizing these combinations demands an in-depth understanding of the virus’s biology, including its modes of replication and potential mechanisms of resistance. Additionally, extensive investigation is necessary to evaluate potential drug–drug interactions, determine ideal dosage regimens, and advance the development of co-formulated medications to enhance patient compliance and treatment outcomes [188].

Furthermore, identifying new antiviral agents with novel mechanisms of action is essential for staying ahead of viral evolution, expanding therapeutic options, improving treatment efficacy and safety, and fostering innovation in antiviral drug development. As an example, PROTAC (Proteolysis Targeting Chimeras) offers an innovative mechanism by utilizing a bifunctional molecule compromising a ligand able to bind the protein of interest (POI) and another component that recruits an E3 ubiquitin ligase, the cellular protein responsible for tagging proteins for destruction [189,190,191,192,193]. When a PROTAC compound binds to a disease-associated protein, it brings the E3 ligase into close proximity, leading to the ubiquitination of the target and its subsequent degradation by the proteasome. This technology offers several benefits compared to traditional therapies. A major advantage is that this approach requires only a selective binder of the POI rather than a functional inhibitor. Consequently, the degrader molecules operate effectively at much lower target affinities compared to conventional inhibitors, potentially reducing susceptibility to point mutations that often compromise drug efficacy with high-affinity ligands [194]. Secondly, the mechanism of PROTAC operates akin to a catalyst in a chemical reaction, where the target protein is degraded without being consumed. This property allows for efficacy at lower doses, thereby mitigating off-target toxicity associated with high-dose drug treatments. Additionally, PROTACs have the ability to disrupt all functions (enzymatic, structural, and scaffolding) of the targeted protein, enabling a pharmacological mechanism that can lead to increased drug potency and may be particularly beneficial in addressing multifunctional targets. However, PROTACs face also challenges related to their molecular properties, including large size and limited water solubility, which can impact their bioavailability and cellular uptake.

While PROTAC technology has been primarily exploited within the anticancer field [195,196,197], its application in the antiviral area is gaining attention [198,199,200,201]. Specifically, PROTAC compounds may be potentially developed against a broad range of viruses by customizing the recruiting ligands to different viral polymerases. This versatility underscores the significant potential of small molecule degraders for creating next-generation antiviral therapeutics that are more durable and effective against a wider range of viral infections [202].

In conclusion, the pursuit of novel small molecules targeting viral polymerases has immense value as they offer a crucial weapon in our fight against viral threats, including emerging and re-emerging infections that pose significant risks to global health [203,204].

## Figures and Tables

**Table 1:** Overview of DNA and RNA viruses treated by approved polymerase inhibitors [2].

	Human Virus, Year of Discovery, Family	Baltimore Classification (Genome Organization)	Polymerase Class and Name	Mode of Transmission	Associated Diseases
**DNA**	HCMV ^1^, 1956 *Herpesviridae*	I (linear, dsDNA)	DdDp, UL54	Blood-borne, bodily fluids, maternal–neonatal	Retinitis, encephalitis, hepatitis, nephritis, mononucleosis-like syndrome, gastroenteritis
	VZV ^2^, 1953 *Herpesviridae*	I (linear, dsDNA)	DdDp, ORF28	Respiratory droplets, Maternal–neonatal	Varicella, Herpes zoster
	HSV ^3^, before 1900 *Herpesviridae*	I (linear, dsDNA)	DdDp, UL30	Sexual or skin contacts, eye, maternal–neonatal	Labialis and genital herpes, keratinitis, pneumonia, encephalitis, meningitis
	HBV ^4^, 1963 *Hepadnaviridae*	VII (circular, (−) ssDNA/dsDNA)	RdDp/DdDp, RT	Blood-borne	Hepatitis B, hepatocellular carcinoma
**RNA**	HCV ^5^, 1989 *Flaviviridae*	IV (linear, (+) ssRNA)	RdRp, NS5B	Blood-borne	Hepatitis C, cirrhosis, and liver cancer
	RSV ^6^, 1957 *Paramyxoviridae*	V (linear, (−) ssRNA)	RdRp, L	Respiratory droplets	Respiratory illness
	SARS-CoV-2 ^7^, 2019 *Coronaviridae*	IV ((+) ssRNA)	RdRp, Nsp12	Respiratory droplets	COVID-19
	HIV ^8^, 1983 *Retroviridae*	VI ((+) ssRNA-RT)	RdRp, RT	Blood-borne, bodily fluids, maternal–neonatal	AIDS
	Flu ^9^, 1933 *Orthomyxoviridae*	VI (linear, (+) ssRNA)	RdDp, Flu Pol	Blood-borne, bodily fluids, maternal–neonatal	Influenza

^1^ Human cytomegalovirus; ^2^ varicella-zoster virus; ^3^ herpes simplex virus; ^4^ hepatitis B virus; ^5^ hepatitis C virus; ^6^ respiratory syncytial virus; ^7^ severe acute respiratory syndrome coronavirus 2; ^8^ human immunodeficiency virus; ^9^ human influenza virus.

**Table 2:** Overview of approved small molecule drugs targeting viral polymerases.

Drug Name (Abbreviation)	Targeted Virus	Biological Activity ^c^	Figure
**Acyclovir (ACV)**	HSV	K_i (HSV-1)_ = 0.08 µM, EC_50 (HSV-1)_ = 0.7 µM [22], EC_50 (HSV-2)_ = 1.6 µM [22]	Figure 5
	VZV	EC_50 (VZV)_ = 3.38 µM [23]	
**Valacyclovir (VACV)**	HSV, VZV	**ACV** prodrug	Figure 5
**Idoxuridine ^a^ (IDU)**	HSV	EC_50 (HSV-2)_ = 2.4 µM [24]	Figure 5
**Trifluridine (TFT)**	HSV	IC_50(HSV-1)_ = 3.07 µM [25]	Figure 5
**Brivudine ^b^ (BVDU)**	HSV VZV	EC_50 (HSV-1)_ = 0.08 µM, EC_50 (HSV-2)_ = 29.0 µM EC_50 (VZV)_ = 0.0844 µM [23]	Figure 5
**Vidarabine ^a^ (VDR)**	HSV VZV	EC_50 (HSV-1)_ = ~10 µM [26] IC_50 (VZV)_ = 5.99 µM [27]	Figure 5
**Ganciclovir (GCV)**	HCMV	EC_50_ = 0.08 µM	Figure 5
**Penciclovir (PCV)**	HSV	K_i (HSV-1)_ = 16.0 µM, K_i (HSV-2)_ = 9.5 µM	Figure 5
**Valganclovir (VGCV)**	HCMV	**GCV** prodrug	Figure 5
**Famciclovir (FCV)**	HSV, VZV	**PCV** prodrug	Figure 5
**Cidofovir (CDV)**	HCMV	K_i_ = 6.6 µM [28]	Figure 5
**Foscarnet (PFA)**	HSV HCMV	EC_50 (HSV-1)_ = 32.6 µM [29] IC_50 (HCMV)_ = 2.5 µM	Figure 5
**Lamivudine ((-)-3TC)**	HBV HIV	IC_50 (HBV)_ = 3.3 µM, K_i (HBV)_ =0.7 µM [30] IC_50 (HIV-1)_ = 0.002 µM	Figure 7
**Tenofovir disoproxil fumarate (TDF)**	HBV HIV	EC_50 (HBV)_ = 0.49 µM EC_50 (HIV-1)_ = 0.0033 µM, EC_50 (HIV-2)_ = 0.0035 µM	Figure 7
**Adefovir dipivoxil (ADP)**	HBV	K_i_ = 0.1 µM	Figure 7
**Tenofovir alafenamide fumarate (TAF)**	HBV HIV	EC_50 (HBV)_ = 0. 0866 µM [31] EC_50 (HIV-1)_ = 0.0035 µM [32], EC_50 (HIV-2)_ = 0.0018 µM [32]	Figure 7
**Telbivudine ^a^ (LdT)**	HBV	IC_50_ = 1.3 µM	Figure 7
**Entecavir (ETV)**	HBV	IC_50_ = 0.0005 µM [33]	Figure 7
**Zidovudine (AZT)**	HIV	IC_50 (HIV-1)_ = 0.1 µM [34], EC_50 (HIV-1)_ = 0.01 µM	Figure 9
**Didanosine ^a^ (ddI)**	HIV	IC_50_ = 0.49 µM [35]	Figure 9
**Zalcitabine ^a^ (ddC)**	HIV	EC_50 (HIV-1)_ = 2.2 µM [36]	Figure 9
**Stavudine ^a^ (d4T)**	HIV	K_i (HIV-1)_ = 0.0083 µM	Figure 9
**Emtricitabine ((-)-FTC)**	HIV	EC_50 (HIV-1)_ = 0.31 µM	Figure 9
**Abacavir (ABC)**	HIV	K_i (HIV-1)_ = 0.021 µM	Figure 9
**Nevirapine (NVP)**	HIV	IC_50 (HIV-1)_ = 0.25 µM	Figure 11
**Delavirdine ^a^ (DLV)**	HIV	IC_50 (HIV-1)_ = 0.021 µM	Figure 11
**Efavirenz (EFV)**	HIV	EC_50 (HIV-1)_ = 0.02 µM	Figure 11
**Etravirine (ETR)**	HIV	IC_50 (HIV-1)_ = 0.0384 µM	Figure 11
**Rilpivirine (RPV)**	HIV	IC_50 (HIV-1)_ = 0.014 µM	Figure 11
**Doravirine (DOR)**	HIV	IC_50 (HIV-1)_ = 0.012 µM	Figure 11
**Ribavirin (RBV)**	HCV, RSV	IC_50 (RSV)_ = 20.9 µM [37]	Figure 14
**Sofosbuvir (SOF)**	HCV	EC_50_ = 0.092 µM [38]	Figure 14
**Dasabuvir ^a^ (DSV)**	HCV	IC_50_ = 0.0028 µM	Figure 14
**Beclabuvir ^b^ (BCV)**	HCV	IC_50_ = 0.003 µM	Figure 14
**Favipiravir (FPV)**	Influenza viruses-A, B, C	EC_50 (influenza A)_ = 0.19 µM EC_50 (H2N2 subtype)_ = 0.083 µM EC_50 (Flu AH3N2)_ = 0.5 µM	Figure 17
**Remdesivir (RDV)**	SARS-CoV-2	EC_50_ = 0.77 µM [39]	Figure 18
**Molnupiravir (MOL)**	SARS-CoV-2	EC_50_ = 0.32–2.66 µM [40]	Figure 18

^a^ Withdrawn from the market and/or no longer recommended for use in the U.S. (https://www.accessdata.fda.gov/scripts/cder/daf/, accessed on 30 March 2024). ^b^ Drugs approved outside U.S. ^c^ Biological activities lacking specific references were sourced from the “NCATS Inxight Drugs” database (https://drugs.ncats.io/, accessed on 30 March 2024).

**Table 3:** Summary of currently available structural data (i.e., PDB IDs) showing a small molecule drug bound to a viral polymerase. In the “Comments” column, MP, DP, and TP describe the phosphorylation state of the co-crystallized drug, specifically indicating whether the small molecule is mono-phosphorylated, di-phosphorylated, or tri-phosphorylated, respectively.

Drug	PDB Ligand Name	PDB IDs	Comments
**Entecavir**	ET9	5XN1, 6IKA, 6KDM	TP state
**Foscarnet**	PPF	5HP1	
**Zidovudine**	AZT	3V4I, 5I42	TP state
	ATM	5HP1, 1N5Y, 1N6Q, 3KLG, 3KLH, 3V4I, 3V6D, 3V81,5I3U	MP state
**Doravirine**	2KW	4NCG, 7Z2H, 7Z2G	
**Stavudine**	D4T	6AMO, 6AN2, 6AN8, 6ANQ, 6ASW, 6AVM, 6AVT, 6WPF, 6WPJ	TP state
	D4M	6WPF	MP state
**Lamivudine**	1RZ	6KDJ, 6KDO, 6OUN, 6UJY	TP state
**Emtricitabine**	43X	6WPH	MP state
	N8G	6UJZ, 6OTZ	TP state, R
	1RY	6OR7, 6UIR, 6UJX, 7LRY	TP state, S
**Nevirapine**	NVP	1FKP, 1JLB, 1JLF, 1LW0, 1LWC, 1LWE, 1LWF, 1S1U, 1S1X, 1VRT, 2HND, 2HNY, 3HVT, 3LP0, 3LP1, 3QIP, 3V81, 4B3Q, 4PUO, 4PWD, 4Q0B, 5HBM, 7KJX, 7Z29, 7Z24	
**Delavirdine**	SPP	1KLM	
**Efavirenz**	EFZ	7KJW, 1IKW, 1IKV, 4B3O, 1JKH, 1FKO, 1FK9, 6BSJ, 6BSI, 6BSH, 6BSG	
**Etravirine**	65B	1SV5, 3M8P, 3MEC, 3MED	
**Rilpivirine**	T27	2ZD1, 2ZE2, 3BGR, 3MEE, 3MEG, 3QLH, 4G1Q, 4ICL, 4ID5, 4IDK, 4IFV, 4IFY, 4IG3, 4KFB, 5CYM, 5CYQ, 6ELI, 7Z2D, 7Z2E, 8DX2, 8DX3, 8DX8, 8DXB, 8DXE, 8DXG, 8DXH, 8DXI, 8DXJ, 8DXK, 8DXL, 8DXM	
**Penciclovir**	HCU	7DOK, 7DOI	MP state
**Tenofovir**	-	1T03	MP state
	TFV	1T05, 3JSM	DP state
**Sofosbuvir**	6SG	4WTG	DP state
**Beclabuvir**	2N7	4NLD	
**Favipiravir**	GE6	7AAP, 7CTT	TP state
	1RP	7DFG	MP state
**Ribavirin**	RBV	3SFU	Unphosphorilated
	RVP	7DFH	MP state
	RTP	2E9R	TP state
**Remdesivir**	F86	7BV2	MP state
	NWX	7UO4	TP state
	-	7L1F, 7B3B, 7B3D, 7B3C, 7D4F, 7C2K, 7BV1, 7BV2	MP state
**Molnupiravir**	-	7OZV, 7OZU	MP state

**Table 4:** Priority emerging diseases declared by the World Health Organization (WHO) (https://www.who.int/activities/prioritizing-diseases-for-research-and-development-in-emergency-contexts; access date: 30 March 2024).

Baltimore Class	Emerging Disease	Polymerase Name	Disease	Available Treatment(s)
**V ((−) ssRNA)**	EboV ^1^	RdRp (L)	Fever, fatigue, muscle pain, headache and sore throat.	General supportive care management. FDA approved two monoclonal antibodies in adults and children.
**IV ((+) ssRNA))**	COVID-19 ^2^	RdRp (nsp12)	Fever, cough, and fatigue; difficulty breathing or mild pneumonia.	
	MERS-CoV ^3^	RdRp (L)	No symptoms or mild respiratory symptoms to severe acute respiratory disease and death	General supportive care management
	SARS ^4^	RdRp (nsp12)	Fever (>38 °C) and sometimes associated with chills, rigors, headache, malaise, and muscle pain.	Supportive treatment based on the symptoms.
	Zikv ^5^	RdRp (NS5)	Fever, rash, conjunctivitis, muscle and joint pain, malaise, or headache.	Treatment of pain and fever with common medicines.
**V ((−) ssRNA))**	CCHF ^6^	RdRp (L)	High fever, back, joint, and stomach pain. Red eyes, flushed face, red throat, and petechiae on the palate are common. In severe cases: jaundice, changes in mood, and sensory perception.	General supportive care management and ribavirin as an antiviral.
	MV ^7^	RdRp (L)	High fever, severe headache and malaise, muscle aches, severe watery diarrhea, abdominal pain, cramping, nausea, and vomiting.	General supportive care management and ribavirin as an antiviral.
	HeV ^8^	RdRp (L)	Respiratory illness with severe flu-like signs and symptoms; encephalitis.	Cases are treated supportively in hospital or in intensive care.
	NiV ^9^	RdRp (L4R)	Encephalitis and can cause mild to severe illness and even death.	Intensive supportive care is provided.
**V(ssRNA)**	LASV ^10^	RdRp (L)	Fever, general weakness, headache, sore throat, muscle, chest pain, nausea, vomiting, diarrhea, cough, and abdominal pain.	No specific treatment; generally supportive therapy.
	RVF ^11^	RdRp (L)	Fever, weakness, back pain, dizziness, ocular disease, encephalitis, or hemorrhagic fever.	Early supportive care. Ribavirin antiviral therapy seems effective.
**Disease X**		A potential **disease** that could cause a serious global emergency.		

^1^ Ebola virus; ^2^ severe acute respiratory syndrome coronavirus 2; ^3^ Middle East respiratory syndrome coronavirus; ^4^ severe acute respiratory syndrome; ^5^ Zika virus; ^6^ Crimean–Congo hemorrhagic fever virus; ^7^ Marburg virus; ^8^ Henipa Virus; ^9^ Nipah virus; ^10^ Lassa fever virus; ^11^ Rift Valley virus.

## Data Availability

Data sharing is not applicable to this article.
